# A novel cervix carcinoma biomarker: Pathological-epigenomics, integrated analysis of MethylMix algorithm and pathology for predicting response to cancer immunotherapy

**DOI:** 10.3389/fonc.2022.1053800

**Published:** 2022-11-02

**Authors:** Yu-Chong Yu, Tian-Ming Shi, Sheng-Lan Gu, Yu-Hong Li, Xiao-Ming Yang, Qiong Fan, Yu-Dong Wang

**Affiliations:** ^1^ Department of Gynecologic Oncology, The International Peace Maternity and Child Health Hospital, School of Medicine, Shanghai Jiao Tong University, Shanghai, China; ^2^ Shanghai Municipal Key Clinical Specialty of Gynecologic Oncology Affiliated to The International Peace Maternity and Child Health Hospital, School of Medicine, Shanghai Jiao Tong University, Shanghai, China; ^3^ Shanghai Key Laboratory of Embryo Original Diseases Affiliated to Shanghai Jiao Tong University School of Medicine, Shanghai, China

**Keywords:** MethylMix, pathomics, cervical squamous cell carcinoma, personal treatment, immunotherapy

## Abstract

Herein, A non-invasive pathomics approach was developed to reveal the methylation status in patients with cervical squamous cell carcinoma and predict clinical outcomes and treatment response. Using the MethylMix algorithm, 14 methylation-driven genes were selected for further analysis. We confirmed that methylation-driven genes were differentially expressed in immune, stromal, and tumor cells. In addition, we constructed a methylation-driven model and explored the alterations in immunocyte infiltration between the different models. The methylation-driven subtypes identified in our investigation could effectively predict the clinical outcomes of cervical cancer. To further evaluate the level of methylation-driven patterns, we constructed a risk model with four genes. Significant correlations were observed between the score and immune response markers, including PD1 and CTLA4. Multiple immune infiltration algorithms evaluated the level of immunocyte infiltration between the high- and low-risk groups, while the components of anti-tumor immunocytes in the low-risk group were significantly increased. Subsequently, a total of 205 acquired whole-slide imaging (WSI) images were processed to capture image signatures, and the pathological algorithm was employed to construct an image prediction model based on the risk score classification. The model achieved an area under the curve (AUC) of 0.737 and 0.582 for the training and test datasets, respectively. Moreover, we conducted vitro assays for validation of hub risk gene. The proposed prediction model is a non-invasive method that combines pathomics features and genomic profiles and shows satisfactory performance in predicting patient survival and treatment response. More interdisciplinary fields combining medicine and electronics should be explored in the future.

## 1 Introduction

Cervical cancer is one of the leading causes of cancer-related deaths in women (1). This disease is most commonly caused by persistent infection with high-risk human papillomavirus (2). Cervical squamous cell carcinoma (CSCC) and cervical adenocarcinoma (CAC) are the two most common histological subtypes, and show differences in carcinogenic mutations, immune microenvironment, treatment response, and clinical outcomes (3–8). Despite advances in prevention and treatment over the past decades, the overall survival rate of patients with localized disease remains below 60%, falling to below 20% if distant metastasis is present (9). Therefore, alternative therapies such as immunotherapy are currently being explored.

Tumor-infiltrating immune cells (TILs) in the tumor microenvironment (TME) play an important role in tumor progression, invasiveness, and therapeutic responses (10, 11). In addition, blocking immune checkpoints, such as PD-1/PD-L1 and CTLA-4, has become a trend in malignancies (12, 13). Since the immune components in the TME inhibit anti-tumor immune responses, most tumors often do not respond to single-agent immunotherapy (14). Therefore, it is necessary to develop excellent biomarkers and to investigate combined therapies to improve immunotherapy efficacy.

DNA methyltransferase catalyzes DNA methylation, an important epigenetic modification that uses S-adenosylmethionine as a donor molecule to add a methyl group to the 5-position of cytosine present in the transcriptional regulatory region of genomic DNA (15, 16). Generally, hypermethylation results in gene silencing, whereas hypomethylation results in gene activation. Hypermethylation of promoter regions in some important genes, such as tumor suppressor genes and DNA repair genes, causes downregulation of their expression, which may lead to abnormal cell differentiation or irreparable DNA damage. Therefore, cancer is believed to be closely associated with hypermethylation (17, 18). DNA methylation is increasingly recognized as a biomarker for assessing cancer risk, facilitating early diagnosis, and predicting prognosis. Therefore, we attempted to combine methylation-targeted therapy and immunotherapy to achieve this goal of tumor treatment.

Pathological sections provide a wealth of information which can be quantified through digital pathology and classical machine learning techniques (19). However, thus far, few digital pathology biomarkers have entered clinical practice, partly due to technical limitations, including complex image analysis algorithms. Previous digital pathology studies used computer-based image analysis methods for cell detection and classification (20), nucleus and mitosis detection (21), microvascular segmentation (22) and other immunohistochemical scoring tasks on histopathological images. Machine learning methods can extract predictors from such images to construct a pathological model.

In this context, we replaced cumbersome genome sequencing with the analysis of pathological features extracted from pathological digital images according to the grouping criteria of the gene models. Using this analysis, we propose a new modeling algorithm to construct pathological features based on methylation-driven genes. Patients with cervical cancer who have this feature can be differentiated in terms of clinical outcomes, tumor infiltration status, and immunotherapy efficacy, which may improve patient management and promote personalized treatment strategies.

## 2 Materials and methods

**Figure 1:**
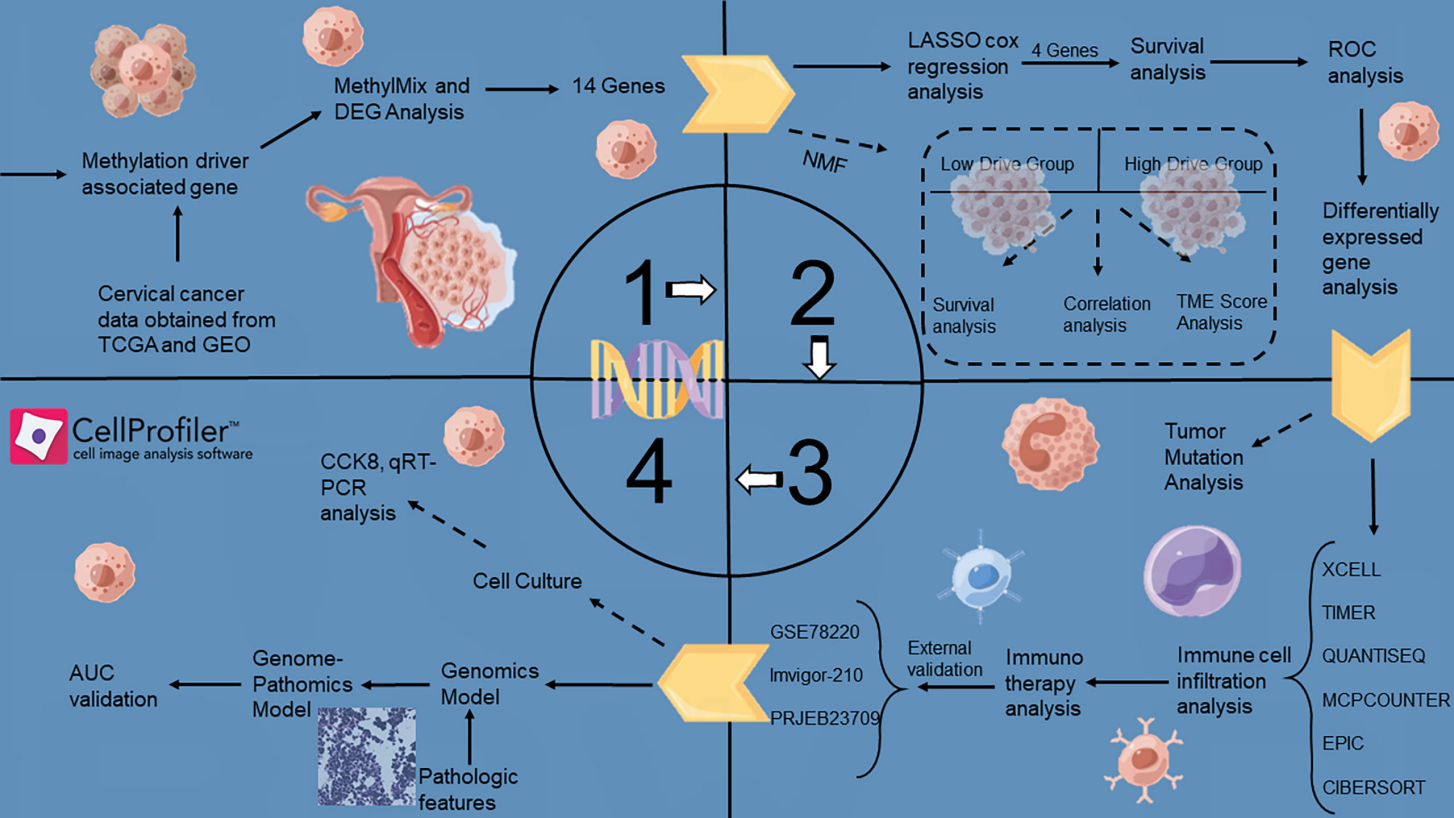
A flowchart of the major steps in this study.

The article landscape was shown in **Figure 1**.

### 2.1 Dataset acquisition and preprocessing

First, the RNA-Seq data and methylation information of CSCC patients were downloaded from the TCGA database. Samples with no survival status or a PFI of< 30 days were excluded from the study. Finally, 243 tumor samples and three normal samples were included. The GSE44001 dataset was downloaded from the GEO database and grouped according to the same inclusion and exclusion criteria as those of the validation cohort. In total, 300 samples were included. Using the “combat” function in the “sva” R package to remove the batch effect on GSE44001 and TCGA-CSCC, the corrected cohort was called a meta-cohort. Three immunotherapy cohorts were included to explore the predictive power of the model for immunotherapy: the GSE78220, Imvigor-210, and PRJEB23709 cohorts. The mutation data (CNVs and SNVs) were processed using the GSCA database. Based on the scRNA-seq data of cervical cancer and normal adjacent tissues in the GSE168652 dataset, genes expressed in at least three cells and cells expressing at least 200 genes were retained. The harmony algorithm was used to merge the different scRNA-seq datasets. The downstream analysis determines the cell clustering based on the corrected harmony embeddings and FindNeighbors functions. Cell clusters were visualized using the t-sne function, and cell types were annotated using the singleR package.

### 2.2 Identification of methylation driven genes

An effective and precise technique called “MethylMix” was used to examine abnormally methylated genes and the relationship between methylation levels and gene expression. First, differential expression analysis was performed based on the “limma” R package in the transcriptome data, with a threshold of | Log2 (fold change) | > 0.2925 and P< 0.05. Differential expression analysis of methylation data revealed a threshold of | Log2 (fold change) | > 0.585 and P-value< 0.05. Subsequently, the above common differential genes were retained, and using the “MethylMix” R package, the β-hybrid model was used to identify the methylation status of genes, which was employed to avoid overfitting according to the Bayesian information standard. Fourteen methylation-driven genes were identified in this study.

### 2.3 Construction of methylation-driven gene-related subtypes

Using the “ConsensusClusterPlus” R package, multiple clusters could be formed using unsupervised consensus clustering based on the k-means computer learning algorithm. Our algorithm used 1000 iterations with 80% sampling of the data for each iteration of the consensus clustering algorithm. The optimal number of clusters was determined using the consensus matrix K value. Two clusters (the low methylation-driven gene drive group and high methylation-driven gene drive group) were selected to assess the sample methylation status.

### 2.4 Construction and validation of methylation-driven prognostic feature

We used the LASSO algorithm to reduce the dimension of 14 methylation-driven genes, followed by multivariate Cox regression analysis to obtain the coefficient of each gene. We established a methylation-driven prognostic feature involving four methylation-driven genes for patients with CSCC. The risk-scoring formula was constructed as follows:


Risk score=Coef i∗Gene i

where Coef i represents the coefficient, and Gene i represents the expression value of each methylated driver gene. Based on the median risk score, we divided the patients into the high- or low-risk groups. More importantly, the K-M survival curve and survival-dependent receiver operating characteristic (ROC) curves for 1-, 3-, and 5-year prognostic values were obtained for the TCGA cohort and strongly validated in the GEO cohort.

### 2.5 Relationship between methylation-driven prognostic feature and immunocyte infiltration

Based on the RNA-seq transcription pattern of CSCC, multiple machine learning algorithms (‘XCELL, TIMER, QUANTISEQ, MCPCOUNTER, EPIC, CIBERSORT’) were used to analyze differences in immunocyte infiltration status between the high- and low-risk groups. In addition, the expression status of common immunoregulators between the high- and low-risk groups is displayed in a box plot. Six immune profile subgroups were identified according to the transcriptome patterns of 33 cancers: wound healing (Immune C1), IFN-gamma dominant (Immune C2), inflammatory (Immune C3), lymphocyte depleted (Immune C4), immunologically quiet (Immune C5), and TGF-beta dominant (Immune C6).

### 2.6 Genomics mutation of risk scoring model

Somatic mutation data were obtained from the TCGA GDC portal (https://portal.gdc.cancer.gov/). We then used the R package “maftools” to draw a waterfall map to depict mutations in high- and low-risk patients.

### 2.7 Screening and construction of digital pathological features

A total of 230 whole-slide imaging (WSI) images were downloaded from the TCGA database for diagnostics. To overcome the problem of oversized digital images, we cut all WSIs into small image blocks (512 × 512 pixels) for non-overlapping sampling at 20x magnification. All patches were color normalized using the Macenko method to obtain a standard normal distribution. Pathological features were extracted using the CellProfiler script published in previous studies. Two pathologists used ImageScope software to annotate the tumor regions (tumor components > 80%) as the region of interest (ROI). Simply put, the UnmixColors, IdentifyPrimaryObjects, MeasureObjectIntensity, Measureobjectsizesshape and MeasureTexture modules separate the color channels of the ROI image and segment the nucleus features to extract pathological features. Finally, high-risk and low-risk classifiers were used to construct a pathological signature using the RF model.

#### 2.7.1 Cell culture

Cell lines HaCaT, Hela, SiHa, and Caski cells were obtained from the American Type Culture Collection. HaCaT, Hela, SiHa, and Caski cells were cultured in DMEM medium containing 10% fetal bovine serum (FBS) and 0.1% penicillin/streptomycin (Fisher Bioreagents, Pittsburgh, Pennsylvania).

#### 2.7.2 Cell viability assay and qRT-PCR analysis

Antibodies to ACSL1 were obtained from Genepharma Corporation (Shanghai, China). Knockdown of ACSL1 expression levels using independent siRNA. Cells transfected with si-ACSL1 in 6-well plates. Twenty-four hours after transfection, against the number of cells, 4,000 cells were seeded into 96-well plates. Cell viability was obtained at the indicated time points using the CCK8 kit. Total RNA was isolated from cells using TRIzol reagent (Invitrogen, Carlsbad, CA, USA). Relative expression was normalized to GAPDH expression with the following primer sequences: ACSL1, forward: 5 ′ -ATC TGC AAG CCA GGA AGA GAG TC-3 ′ and reverse: 5 ′ -CTT GCT TGA TGC TTT GGT CTG T-3 ′; GAPDH forward: 5 ′ -CAT CAC CAT CTT CCA GGA GCG-3 ′ and reverse: 5 ′ -TGA CCT TGC CCA GCC TTG-3 ′.

### 2.8 Statistical analysis

Continuous data with a normal distribution were compared using independent Student’s t-test. For comparisons between subgroups, Kaplan–Meier analysis with the log-rank test was used. All statistical analyses were performed using the R software (version 4.0.3). Statistical significance was set at P< 0.05

## 3 Results

### 3.1 Identifying methylated differentially genes in cervical cancer

Methylation differential genes (MDGs) and differentially expressed genes (DEGs) were identified using R-package Limma and edge, respectively. MDG and DEG were recombined into normal methylation group, methylation cancer group and gene expression cancer group. Through the mixed model and Wilcoxon rank test, 20 MDGs were found. A heatmap was generated using the R package based on the methylation levels of 20 MDGs. In addition, the correlation diagram and methylation distribution diagram were shown in **Supplementary Figure 1A** and **Table S1**. There are only 14 MDGs with methylation regulation. We further explored these 14 genes.

### 3.2 Genome mutation landscape of candidate genes

**Figure 2:**
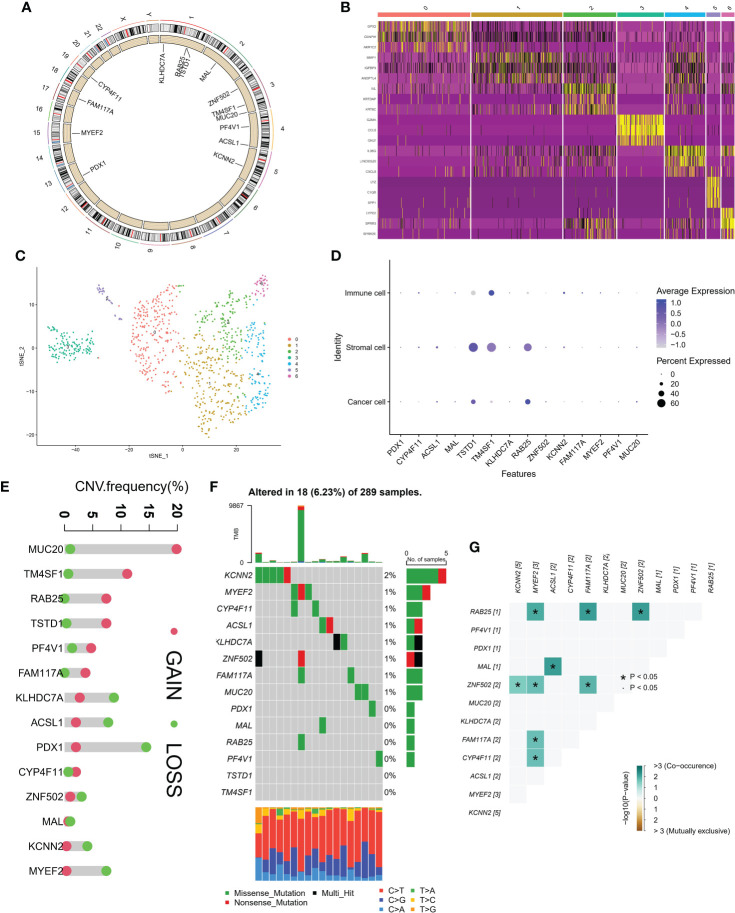
Genomic landscape of methylation driven genes. **(A)** Localization of methylation driven genes in chromosomes. **(B)** Expression levels of methylation driven genes in seven cell subsets. **(C)** Spatial distribution in seven cell subsets. **(D)** Average expression of methylation driven genes in three cell subsets. **(E)** CNV frequency of methylation driven genes. **(F)** tumor Mutation burden of methylation driven genes in samples. **(G)** Interaction between methylation driven genes.

**Figure 2A** showed the location of 14 MDGs in chromosomes, BAB25, TSTD1 on chromosome 1, MAL on chromosome 2, ZNF502, TM4SF1, MUC20 on chromosome 3, PF4V1, ACSL1 on chromosome 4, KCNN2 on chromosome 5, PDX1 on chromosome 13, MYEF2 on chromosome 15, FAM117A on chromosome 17, CY4F11 on chromosome 19. Based on single cell sequencing data, we identified 7 cell subsets. The heatmap showed the expression patterns of 14 MDGs in 7 cell subsets (**Figure 2B**). The tSNE algorithm displayed the distribution of seven cell subsets (**Figure 2C**). We found that TSTD1 and RAB25 were highly expressed in stromal cells and immune cells, and lowly expressed in immune cells. TM4SF1 was highly expressed in both immune cells and stromal cells, and the average expression value was higher in immune cells (**Figure 2D**). In addition, based on the GSCA database, we observed the mutations of 14 MDGs (CNV and SNV). We found that MUC20 had the highest CNV frequency, mainly GAIN, and the highest LOSS was mainly PDX1 (**Figure 2E**). Based on the data of 289 samples, the overall mutation rate of 14 MDGs was 6.23%, and the general frequency of KCNN2 was the highest, followed by MYEF2 and CYP4F11(**Figure 2F**). In addition, the correlation between MDGs was performed in R tool. MYEF2 was associated with four genes, RAB25, ZNF502, FAM117A, CYP4F11, respectively. Among them, it was positively correlated with RAB25, and the remaining correlation was shown in **Figure 2G**.

### 3.3 Prognostic performance of candidate genes

**Figure 3:**
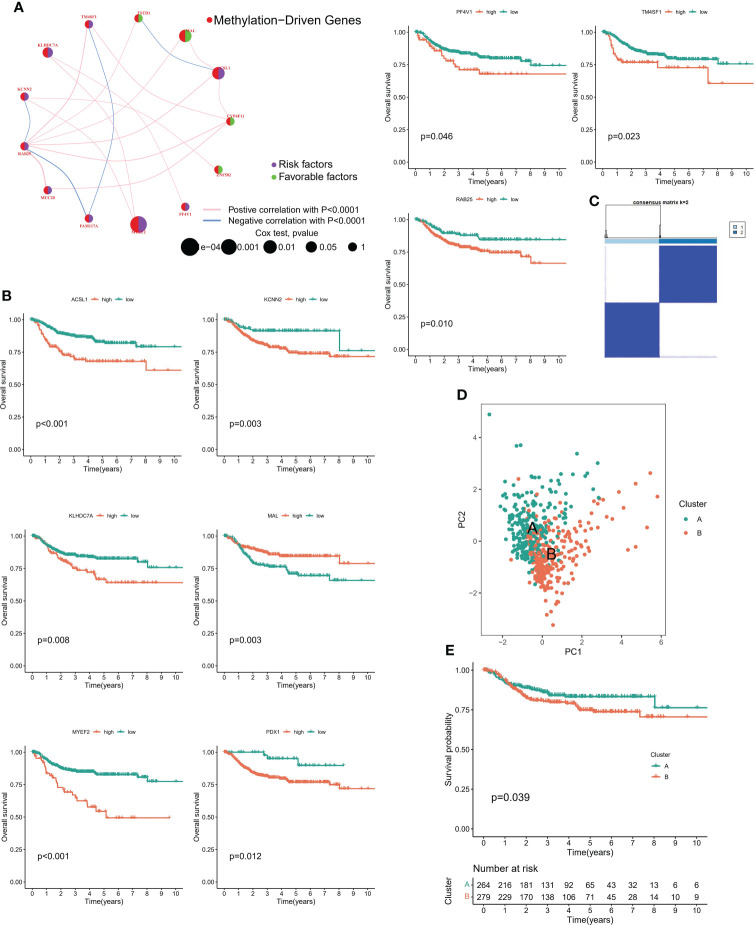
Methylation-driven associated subtypes. **(A)** Network diagram showing the prognostic significance and relevance of methylation-driven genes. **(B)** Prognostic value of methylation driven genes. **(C)** Consensus Matrix for unsupervised cluster analysis. **(D)** PCA analysis showed the distribution of different subtypes. **(E)** K-M curves showed the clinical outcomes among different subtypes.

**Figure 4:**
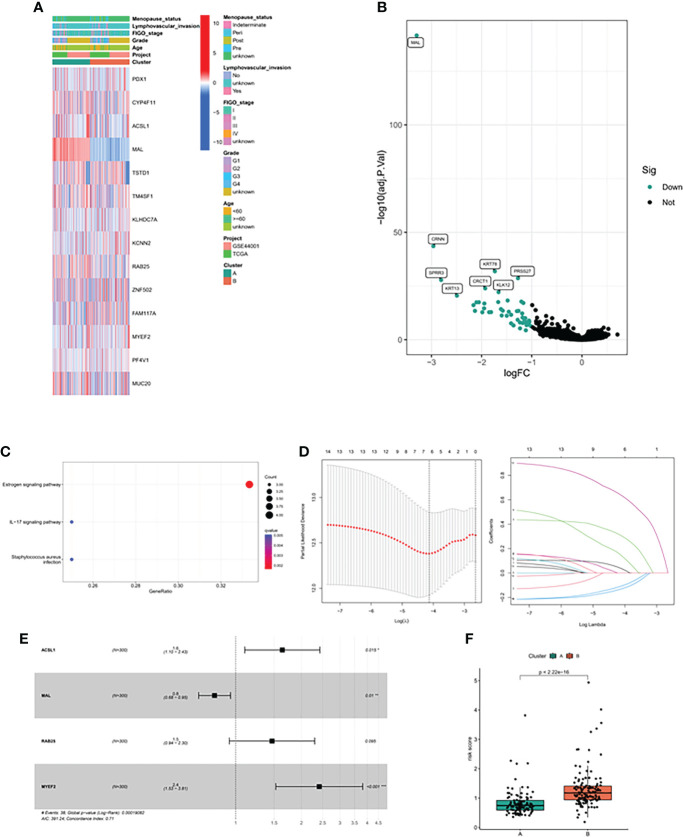
Methylation driven associated predictive model. **(A)** Heatmap of methylation driver genes among different clinical subtypes and methylation driven related subtypes. **(B)** The volcano map showed the differentially expressed genes between the two subtypes. **(C)** GO analysis of differentially expressed genes. **(D)** LASSO algorithm was used to reduce the correlation between methylation driven genes. **(E)** The Forest map displayed the hazard ratios of the 4 target genes. **(F)** Risk score between the two subtypes.

Firstly, we constructed a network diagram to observe the correlation and genetic properties of 14 MDGs. Only TSTD1, MAL, CYP4F11 and ZNF502 were favorable factors, and the rest were risk factors. The blue line connection part represented a negative correlation between the two genes, while the red line represented a positive correlation (**Figure 3A**). **Figure 3B** displayed the K-M survival curve of each MDGs. Among the 14 MDGs, 9 genes had the ability to predict the clinical prognosis of cervical cancer. In addition, Except for the MAL gene, which was served as a tumor suppressor gene, all the others were oncogenic genes. The lower the level of MAL gene, the worse the prognosis of cervical cancer patients (**Figure 3B**). Then we divided the patients into 2 subgroups according to the expression patterns of 14 MDGs genes (**Figure 3C**). The PCA algorithm presented the distribution of each sample. We can clearly find that the patients in cluster A and cluster B subgroups were in different positions, suggesting that the unsupervised clustering effect was significant (**Figure 3D**). The K-M survival analysis showed that patients with cluster A had better survival status (**Figure 3E**). The heatmap showed the expression levels of 14 genes in different subgroups and clinicopathological features (**Figure 4A**). The ' CIBERSORT ' algorithm was used to assess the levels of infiltration of 22 immune cells between different subgroups, with higher levels of immune effector cells in patients in cluster A (**Supplementary Figure 1C**). We performed differential expression analysis on samples of cluster A and cluster B, and **Figure 4B** displayed the differentially expressed genes. The GO pathway analysis was performed to identify biological pathway of differentially expressed genes, and the estrogen signaling pathway was highly correlated with these genes (**Figure 4C**). Subsequently, the LASSO regression algorithm was conducted to obtained independent predictors from 14 MDGs (**Figure 4D**), then multivariate regression analysis screened prognostic genes from these genes. The tree diagram showed four independent prognostic factors, ACSL1, MAL, RA25, MYEF2. Among them, MAL was a protective factor, and the rest were cancer-promoting factors (**Figure 4E**). We constructed a risk model for these four prognostic genes. **Figure 4F** showed that the risk score of patients with cluster A was lower.

### 3.4 Validation of risk scoring model

**Figure 5:**
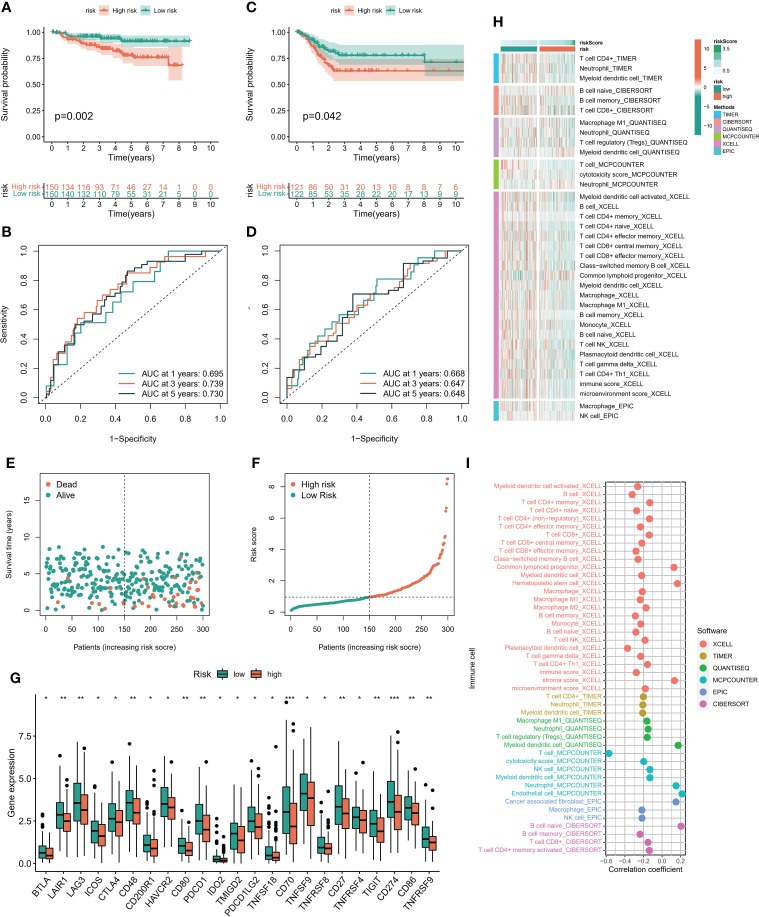
Immune cell infiltration landscape. **(A)** The K-M survival model was constructed to explore the predictive performance in TCGA cohort. **(B)** The ROC curve was conducted to verify the predictive performance of predictive model in TCGA cohort. **(C)** The K-M survival model was constructed to explore the predictive performance in GEO cohort. **(D)** The ROC curve was conducted to verify the predictive performance of predictive model in GEO cohort. **(E, F)** Distribution of risk score and survival status of patients with cervical cancer. **(G)** Expression of immunomodulators between high and low risk groups. **(H)** Heatmaps constructed by six immune infiltration analysis algorithms were employed to assess the level of cell infiltration among each subpopulation. **(I)** Correlation between immune cell infiltration level and riskscore. *P < 0.05, **P < 0.01, ***P < 0.001.

In order to verify the methylation-related subtypes and the difference of immune landscape, K-M survival curve and ROC curve were conducted to observe the performance of prognostic models, and multiple machine learning algorithms ‘ XCELL, TIMER, QUANTISEQ, MCPCOUNTER, EPIC, CIBERSORT ‘ were performed to evaluate the difference of immune landscape. The survival prognosis of the high-risk group was significantly worse than that of the low-risk group. The 1-, 3-, and 5-year predictive performance of the prediction model was 0.695, 0.739, and 0.730 in TCGA cohort, respectively (**Figures 5A, B**). The above trends were consistent in the GEO cohort (**Figures 5C, D**). **Figures 5E, F** showed the distribution of risk scores and survival status of patients in the high and low risk groups. There were significant differences in the expression level of immunomodulators and immune cell infiltration between the low-risk group and the high-risk group. Patients in the low-risk group had remarkably higher expression of immunomodulators, such as CTLA4, CD80, PDCD1, etc. (**Figure 5G**). In addition, immune cells were mainly enriched in the low-risk group, and the level of immune cell infiltration was negatively correlated with risk score (**Figures 5H, I**).

### 3.5 Immunotherapy prediction feasibility certification

**Figure 6:**
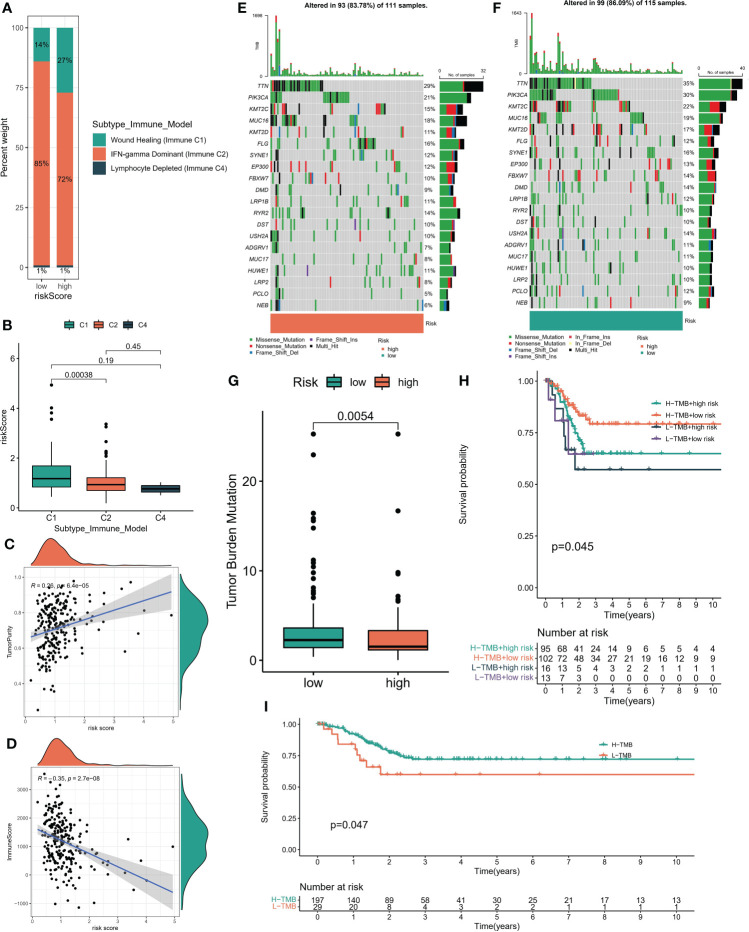
**(A)** Proportion of immune subsets among different risk subgroups. **(B)** Riskscore of immune subsets. **(C, D)** Correlation between risk score and StromalScore, ImmuneScore which obtained by “ESTIMATE” algorithm. **(E, F)** Global mutation landscape of high and low risk subgroups. **(G)** Tumor mutation burden score for high - and low-risk subsets. **(H)** The K-M algorithm was used to evaluate the predictive performance of the risk model +TMB model. **(I)** K-M algorithm was used to evaluate the prediction performance of TMB model.

**Figure 7:**
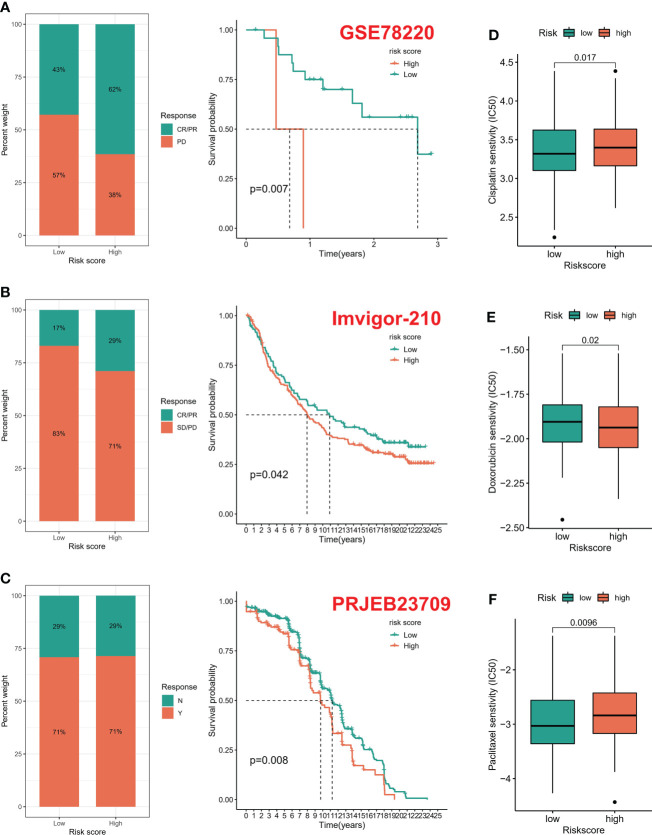
Drug susceptibility prediction. **(A–C)** Treatment effect prediction in three immunotherapy cohorts (GSE78220, IMVigor-210, PREB23709). **(D–F)** Chemotherapeutic drug sensitivity prediction.

According to the six immune subtypes in the previous study, we compared the proportion of different immune subtypes in the high and low risk groups. We found that there was a total of three immune subtypes in cervical cancer patients. Immune C2 accounted for 85% and Immune C1 accounted for 14% in low-risk patients, while Immune C2 accounted for 72% and Immune C1 accounted for 27% in high-risk patients (**Figure 6A**). In addition, the Immune C1 subtype had the highest risk score and the Immune C4 subtype had the lowest risk score (**Figure 6B**). Subsequently, the ESTIMATE algorithm showed that the risk score was positively correlated with the TumorScore and negatively correlated with the ImmuneScore, which meant that patients with high-risk scores were more malignant and had lower anti-tumor immune effects (**Figures 6C, D**). The overall mutation rate of patients with low-risk score was higher than that of patients with high-risk score, 86.09% and 83.78% respectively (**Figures 6E–G**). We combined the risk score and TMB index to predict the clinical prognosis of patients with cervical cancer. The prognosis of patients with L-TMB + high risk was the worst, and the survival time of patients with H-TMB + low risk was the longest (**Figure 6H**). In addition, the survival time of patients with H-TMB was longer than that of patients with L-TMB (**Figure 6I**). We obtained three immunotherapy cohorts, GSE78220, Imvior-210, PRJB23709 cohorts. We found that the high-risk group had a significant effect on immunotherapy, and the survival status of patients in the low-risk group was better than that in the high-risk group (**Figures 7A–C**). In terms of chemotherapy, patients in the low-risk group were more sensitive to Cisplatin and Paclitaxel, while patients in the high-risk group were more sensitive to Doxorubicin (**Figures 7D–F**).

### 3.6 Screening and construction of digital pathological features

**Figure 8:**
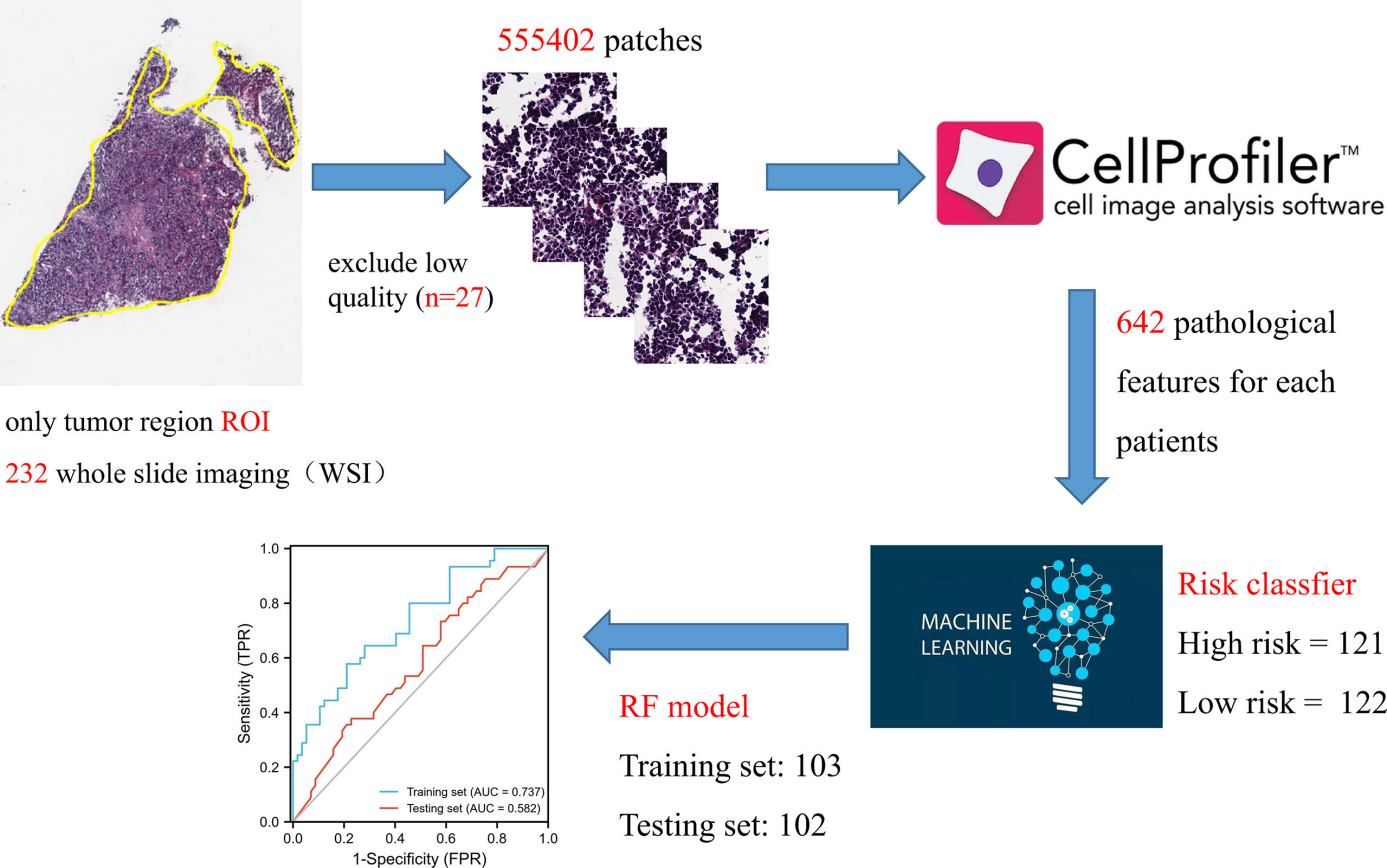
Schematic representation of pathogenomic construction.

The constructed genome risk score model has great predictive performance, but due to too many parameters and expensive sequencing cost, we attempt to replace the genome model with pathological WSI. A total of 232 WSIs containing tumor tissues were obtained from the TCGA database, of which 27 low-quality WSIs were excluded, and 205 samples were finally included. Pathological features were extracted based on the CellProfiler script published in previous studies. Two pathologists used ImageScope software to annotate tumor regions (tumor components > 80%) as ROI. In simple terms, the UnmixColors, IdentifyPrimaryObjects, MeasureObjectIntensity, Measureobjectsizesshape and Measure Texture modules were conducted to separate the color channels of the ROI image and segment the nuclear features. Finally, each WSI generates 642 pathological features. RF model was employed to construct pathological feature signature with high/low risk as classifier. The prediction performance of training set is 0.737, and that of testing set is 0.582 (**Figure 8**).

### 3.7 *In vitro* assays

By reviewing previous references, it was found that ACSL1 was an unexplored gene in cervical cancer. Hence, we selected ACSL1 as the candidate molecule to perform cell function assays. Real-time qPCR analysis indicated that ACSL1 was significantly upregulated in three CC cell lines (**Figure S2A**). The transfection epitranscriptomics of three shRNAs were detected, which revealed that sh-ACSL-2 presented the highest transfection efficiency in Caski cell lines (**Figure S2B**). Similarly, the CCK-8 assay also showed that the viability of Caski was most significant suppressed after transfection of sh-ACSL-2 (**Figure S2C**).

## 4 Discussion

Currently, the staging methods of the International Federation of Gynecology and Obstetrics (FIGO) are the most commonly used for cervical cancer (23). Imaging or pathological examination are the major components of the FIGO staging system. In some nonsurgical patients, staging was based on imaging and doctors’ subjective judgments. This is inherently inaccurate if there is inflammatory disease of the pelvis, endometriosis, or obesity. There is a significant difference in the prognosis of patients with FIGO staging due to the significant difference between the clinically determined staging and surgical pathological results (24). With the rapid development of cancer biology knowledge and the discovery of biological profiles to predict cancer outcomes and treatment responses, oncologists are increasingly using a variety of related, non-anatomic (including molecular) factors to predict the prognosis of individual patients. As such, there is an urgent need for in-depth research to identify effective biomarkers for the early detection and treatment of cervical cancer.

Numerous recent studies have demonstrated that hypomethylation results in gene overexpression, whereas hypermethylation causes low gene expression, which plays an important role in the occurrence and progression of various tumors (25, 26). It is possible that abnormally methylated genes can cause gene expression disorders, transcriptional disorders, and abnormal differentiation of cells (27). Jiao et al. previously provided evidence that SEPT9 methylation may be a biomarker for the diagnosis of cervical cancer, which functions by promoting tumorigenesis and radiation resistance of cervical cancer by targeting the HMGB1-Rb axis, and induces macrophage polarization by mediating Mir-375. With this knowledge, Jiao suspected that SEPT9 may be a potential screening and therapeutic biomarker for cervical cancer (28). Kremer et al. reviewed the role of host cell gene hypermethylation in tumorigenesis and the progression of cervical cancer, and discussed the potential clinical application of methylation analysis in the management of high-risk HPV (hrHPV)-positive women. They suggested that methylation testing may be useful for: 1) Classification of women with high risk HPV types to detect cervical cancer and advanced cervical intraepithelial neoplasia; 2) as a secondary classification of women with minor cytological abnormalities to identify women at risk of CIN 3 or more; 3) as an exit test for women dropping out of a screening program to identify cervical cancer and advanced CIN; and 4) support for CIN management (29). Methylation plays a vital role in cervical cancer progression.

In our investigation, the MethylMix algorithm was used to identify MDGs in cervical cancer and construct a mixed model. For this step, standardized methylation and gene expression data were employed as input matrices. Based on the gene expression and related methylation levels, 20 DMGs were identified. Next, we analyzed the relationship between the 20 DMGs and the prognoses. A combination of clinical information, gene expression, and methylation data were acquired from the TCGA dataset. A set of signatures based on LASSO-COX regression analysis was constructed. It is worth noting that although dysregulation of some of these genes in tumors has been studied, their methylation levels have rarely been mentioned. Therefore, our genomic model combines methylation information with genomic data to predict clinical outcomes and treatment strategies in patients with cervical cancer. In addition, the prediction model is externally validated, which provides a new method for the prognostic assessment of cervical cancer.

In addition, we identified six immune profile subgroups based on transcriptome patterns in 33 cancers: wound healing (immune C1), IFN-γ dominant (immune C2), inflammation (immune C3), lymphocyte depletion (immune C4), immune quietness (immune C5), and TGF-β dominant (immune C6). Histological and immunophenotypic classification of cervical cancer by expression of the p53 homolog p63 has been performed in a pathological study, which is similar to our study (30). It has been shown that IFN-γ gene polymorphisms may contribute to cervical cancer susceptibility, and this result helps to support IFN-gamma dominant (Immune C2) in our immunophenotyping (31). Inflammation following viral infection is a driving force that accelerates cancer development. Infiltrating immune cells and their secreted cytokines will greatly contribute to the malignant features of cervical cancer. A better understanding of the mechanisms involved in inflammation and cancer progression will lead to innovative approaches for treating cervical cancer (32).

In addition, it has also been shown that there is an association between common low-penetrance alleles in the TGFB signaling cascade and altered cervical cancer risk in women, a result that will underpin our immunophenotyping C6, and “immunotherapy” representing cervical squamous cell carcinoma is prognostic (33, 34).

For the link between DNA methylation and pathology, this can be understood in terms of the distinction between DNA methylation in cellular physiological and pathological conditions. DNA methylation is one of the epigenetic mechanisms regulating gene expression (35). In normal cells, a significant degree of methylation is characteristic of extragenic DNA (cytosines within CG dinucleotides) (36). Changes in methylation patterns, which may emerge with the age of the organism and cancer development, have been observed in three regions of exon 5 of the p53 gene in non-small cell lung cancer (36, 37). With the application of real-time PCR technology (using primers for methylated and unmethylated sequences), we have been able to find new markers for early detection of cancer.

Our study still has some limitations. Although methylation profiles are advantageous because genomic models require high sequencing costs and stringent sample storage conditions, we try to replace this with clinical WSI data. However, the images of pathomics may lead to differences in HampE staining results due to different staining methods and raw materials in different places. In addition, since data from our genomic model came from TCGA, we could identify each patient ‘s pathohistological features based on their unique ID in the TCGA platform. So, we can link genomic models to pathomics models. However, we still fail to clarify the specific molecular mechanisms underlying the relationship between genomics and pathology, which require further exploration. In addition, clinical risk factors such as pathological subtype, gender, age, and stage could be considered in the model in the future. Finally, further experiments and studies are needed to understand developmental mechanisms and investigate effective treatments for cervical cancer.

## Data availability statement

The original contributions presented in the study are included in the article/**Supplementary Material**. Further inquiries can be directed to the corresponding authors.

## Author contributions

Y-CY conceived and designed the investigation. Y-DW and QF provided expert advice in the study, performed editing and proofreading of the manuscript. All authors contributed to the study and approved the submitted version.

## Funding

This study is supported by The Shanghai Municipal Key Clinical Specialty (No. shslczdzk06302), National Natural Science Foundation of China (No. 82103029), The Project of the Science and Technology Commission of Shanghai Municipality (No. 21ZR1469500), The Shanghai Jiao Tong University Medicine-Engineering Fund (No. YG2021QN137)

## Conflict of interest

The authors declare that the research was conducted in the absence of any commercial or financial relationships that could be construed as a potential conflict of interest.

## Publisher’s note

All claims expressed in this article are solely those of the authors and do not necessarily represent those of their affiliated organizations, or those of the publisher, the editors and the reviewers. Any product that may be evaluated in this article, or claim that may be made by its manufacturer, is not guaranteed or endorsed by the publisher.
