# Supplementary material for: A novel cervix carcinoma biomarker: Pathological-epigenomics, integrated analysis of MethylMix algorithm and pathology for predicting response to cancer immunotherapy
Source: Front Oncol. 2022 Nov 2;12:1053800. doi: 10.3389/fonc.2022.1053800 (PMC9667097; doi:10.3389/fonc.2022.1053800)
Supplement: Supplementary file 1 [file DataSheet_1.zip › raw data for editor checking/Fig2/7_cluster_heatmap.pdf]

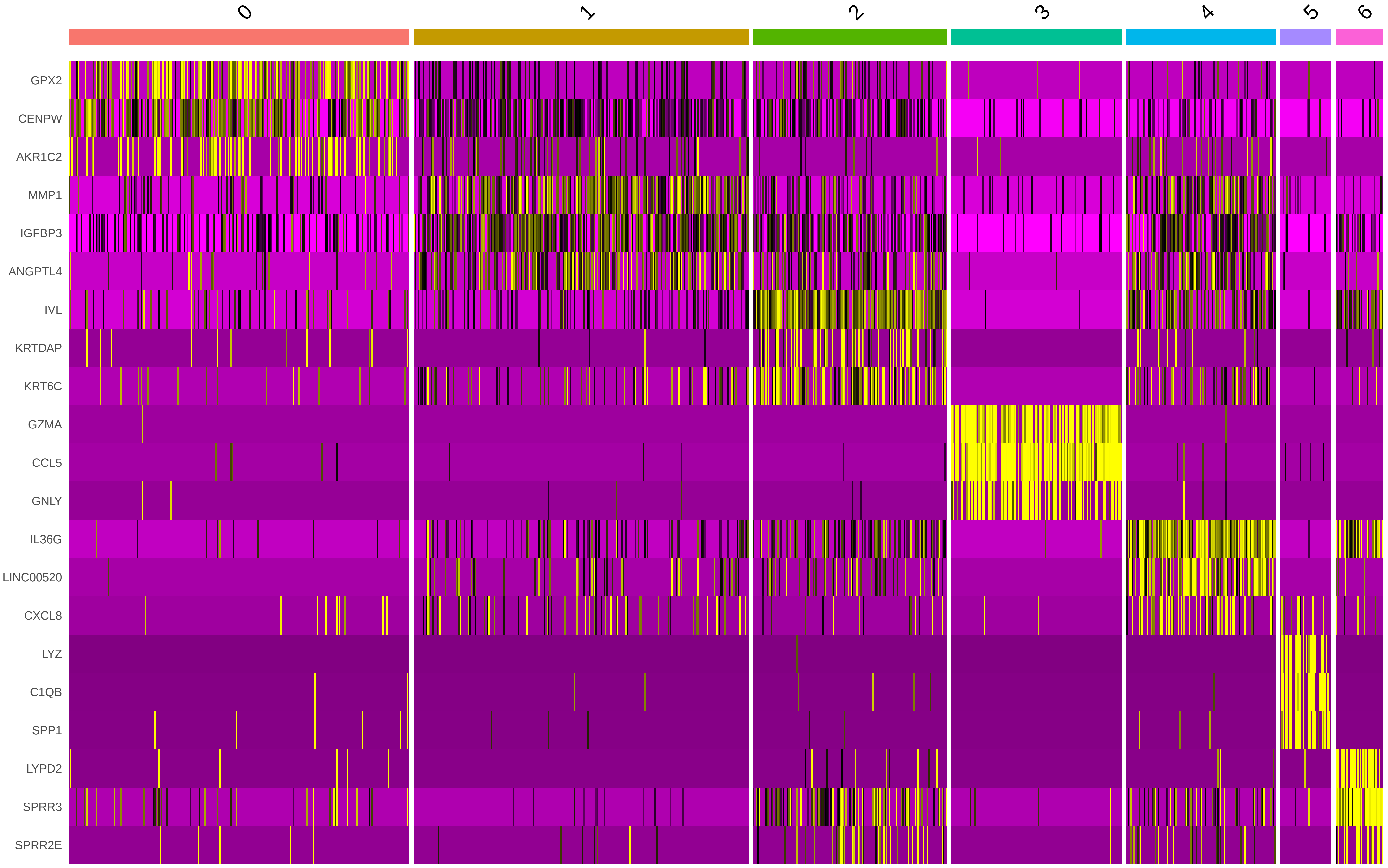

0
1
2
3
4
5
6
GPX2
CENPW
AKR1C2
MMP1
IGFBP3
ANGPTL4
IVL
KRTDAP
KRT6C
GZMA
CCL5
GNLY
IL36G
LINC00520
CXCL8
LYZ
C1QB
SPP1
LYPD2
SPRR3
SPRR2E
