# Supplementary figures and images for: A novel cervix carcinoma biomarker: Pathological-epigenomics, integrated analysis of MethylMix algorithm and pathology for predicting response to cancer immunotherapy
Source: Front Oncol. 2022 Nov 2;12:1053800. doi: 10.3389/fonc.2022.1053800 (PMC9667097; doi:10.3389/fonc.2022.1053800)

**0.94**

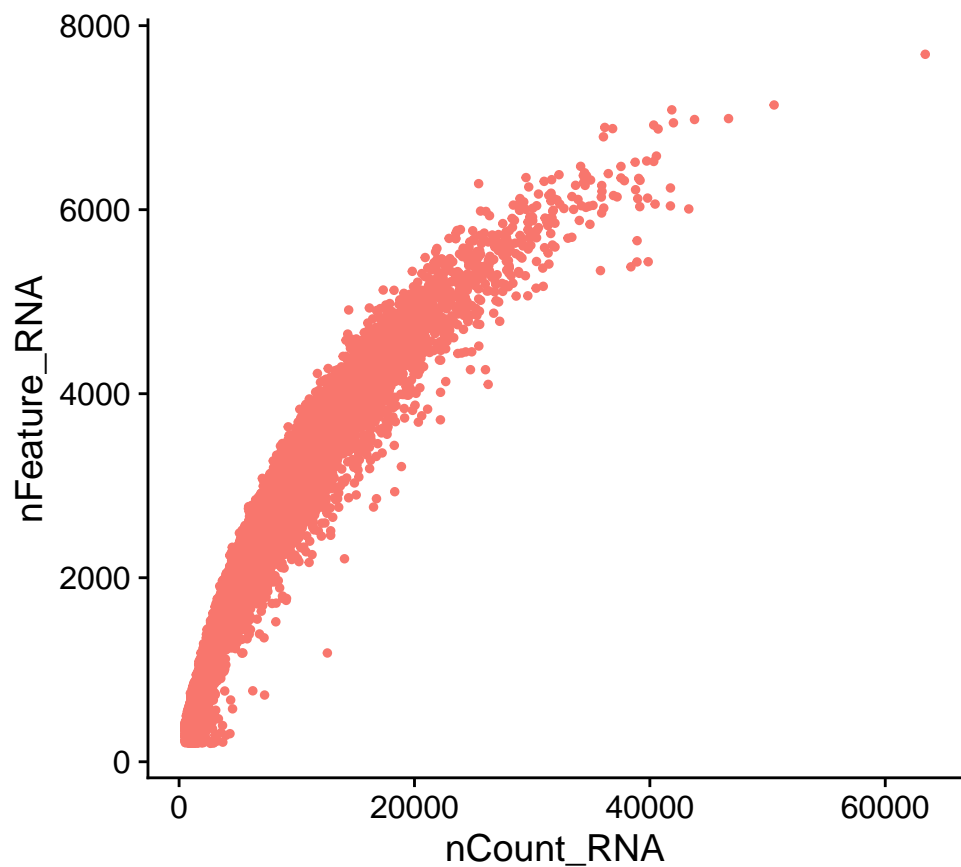

**0.01**

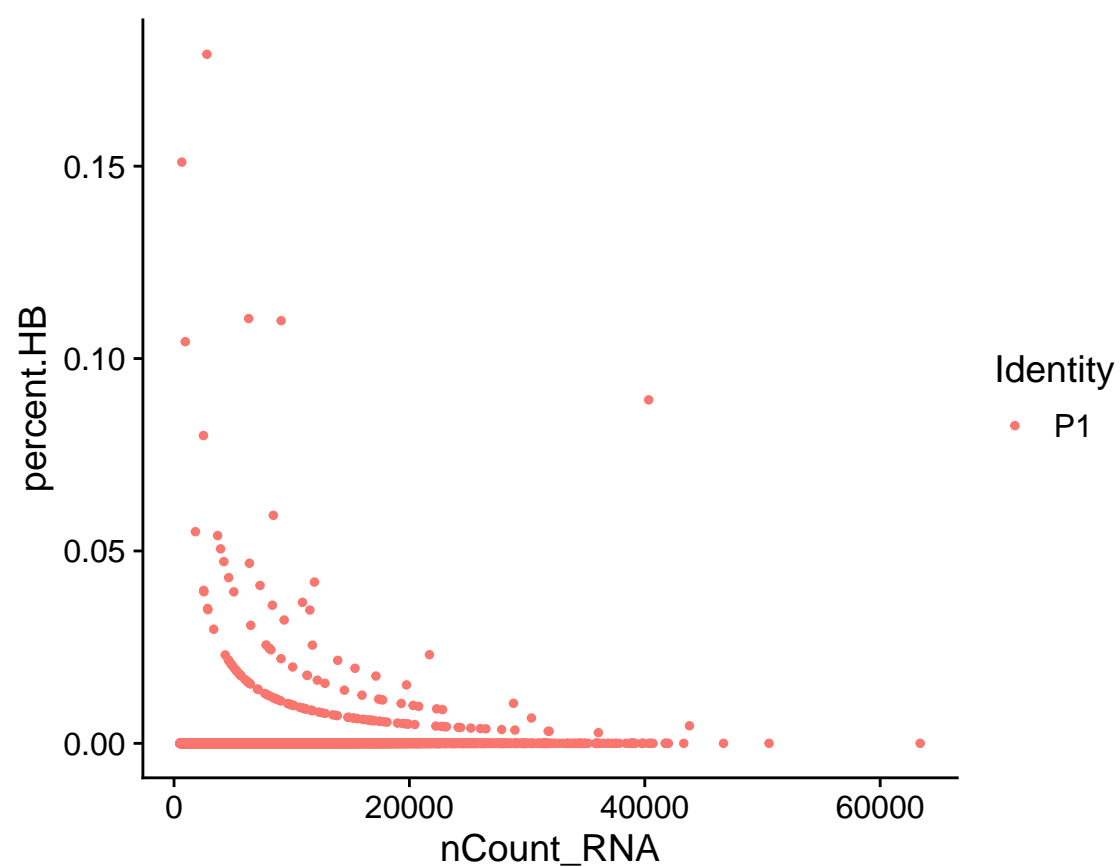

**-0.23**

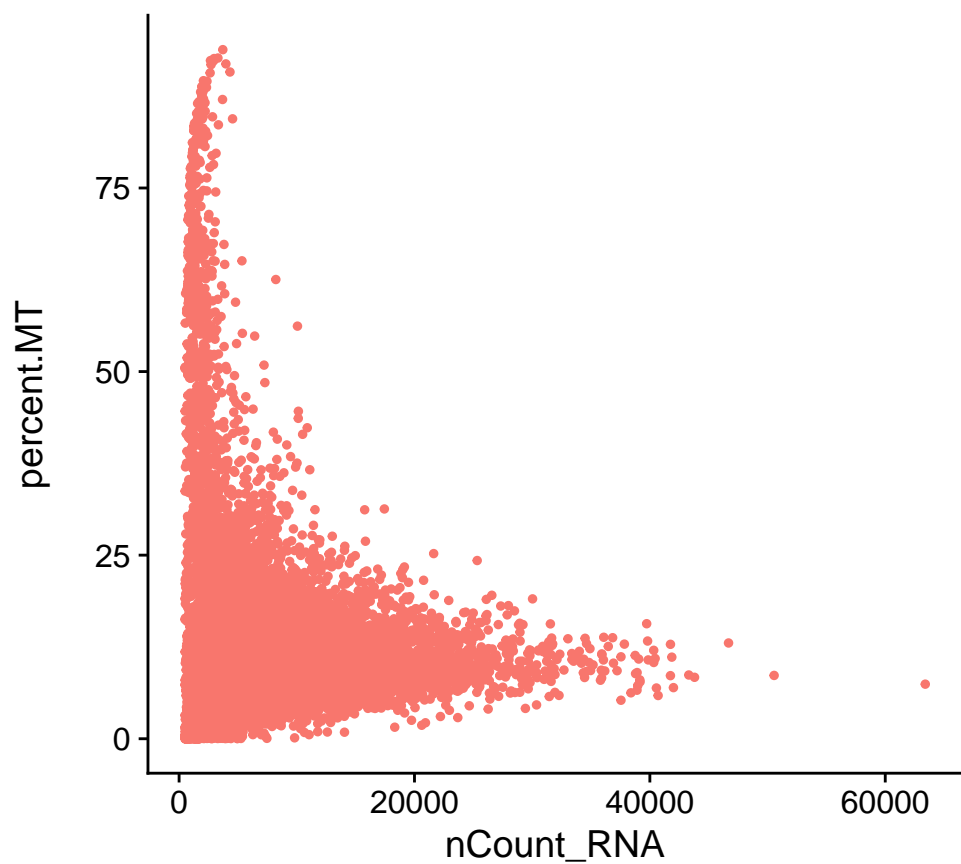

**0.24**

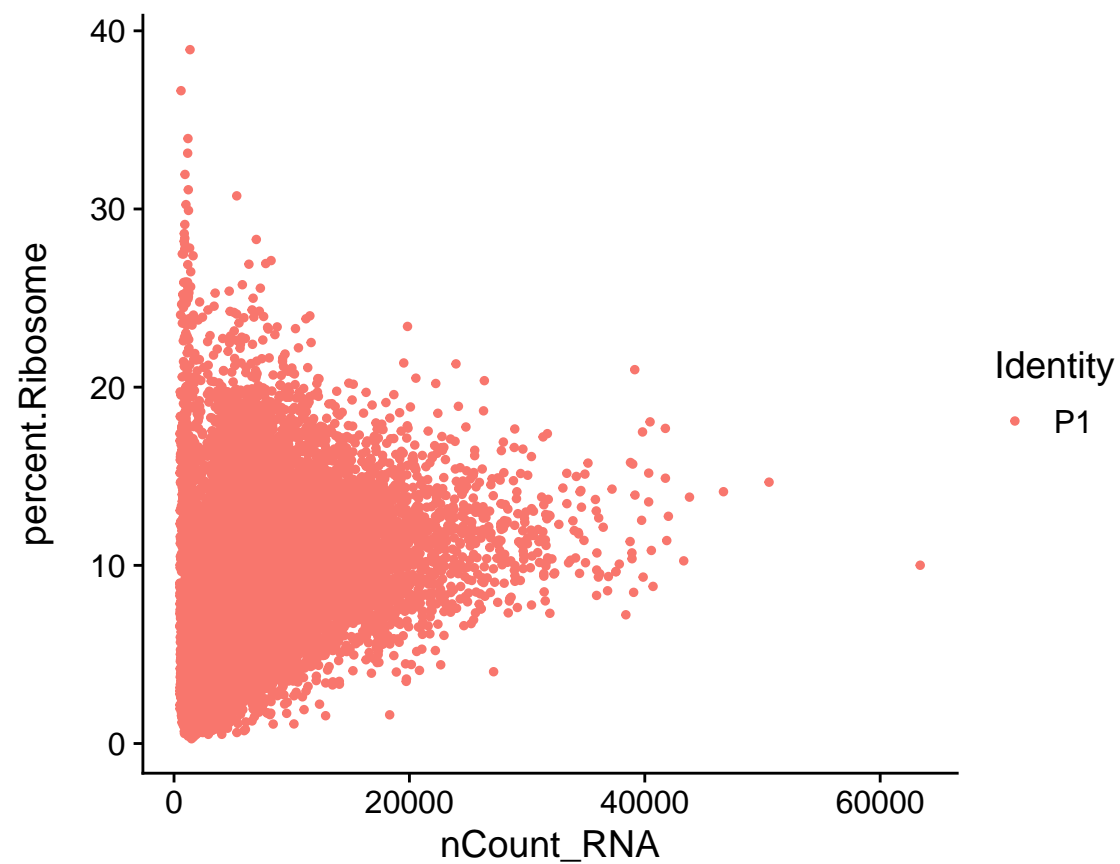

Supplement: Supplementary file 1 [file DataSheet_1.zip › raw data for editor checking/Fig2/1_feature_relationships.pdf]

**nFeature\_RNA**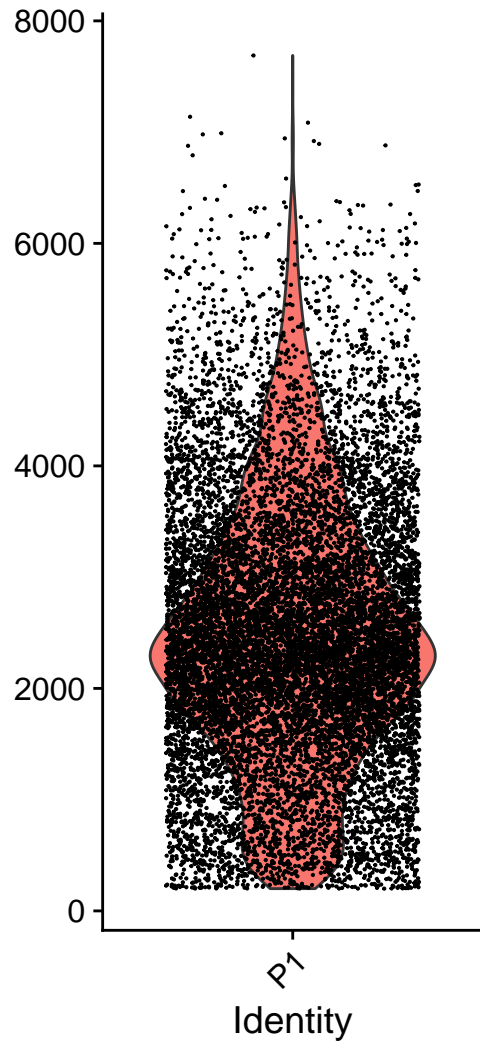**nCount\_RNA**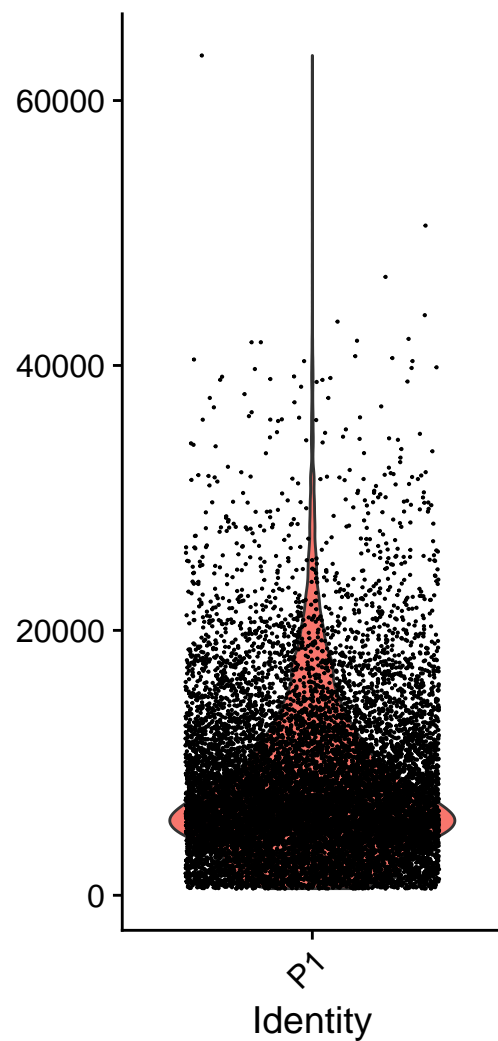**percent.HB**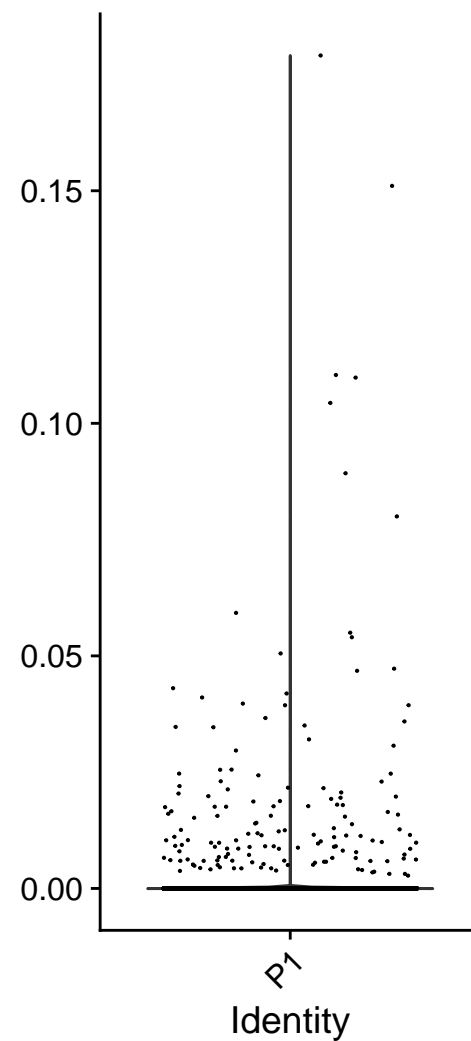**percent.MT**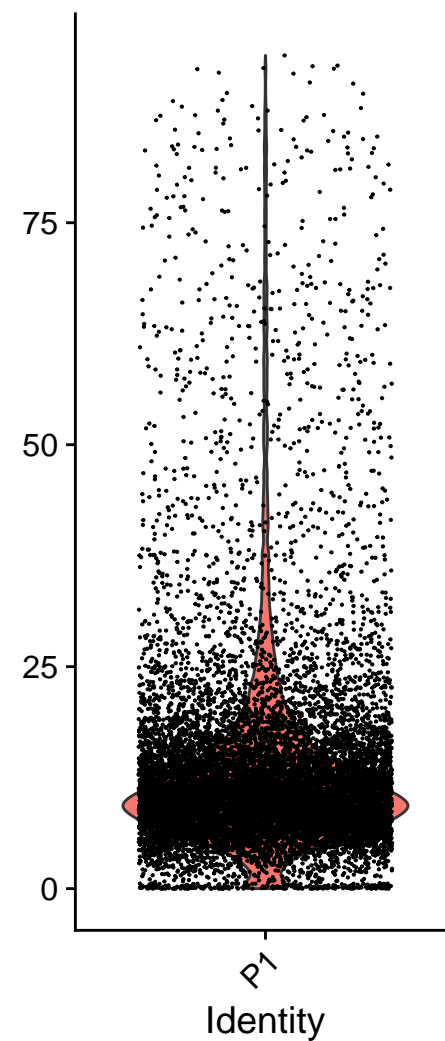**percent.Ribosome**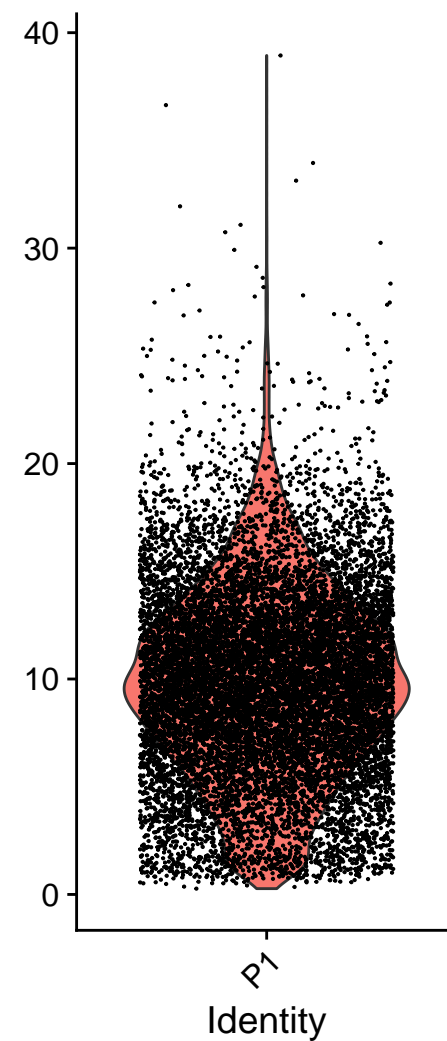

Supplement: Supplementary file 1 [file DataSheet_1.zip › raw data for editor checking/Fig2/1_feature_UMI_MT_HB_Ribosome.pdf]

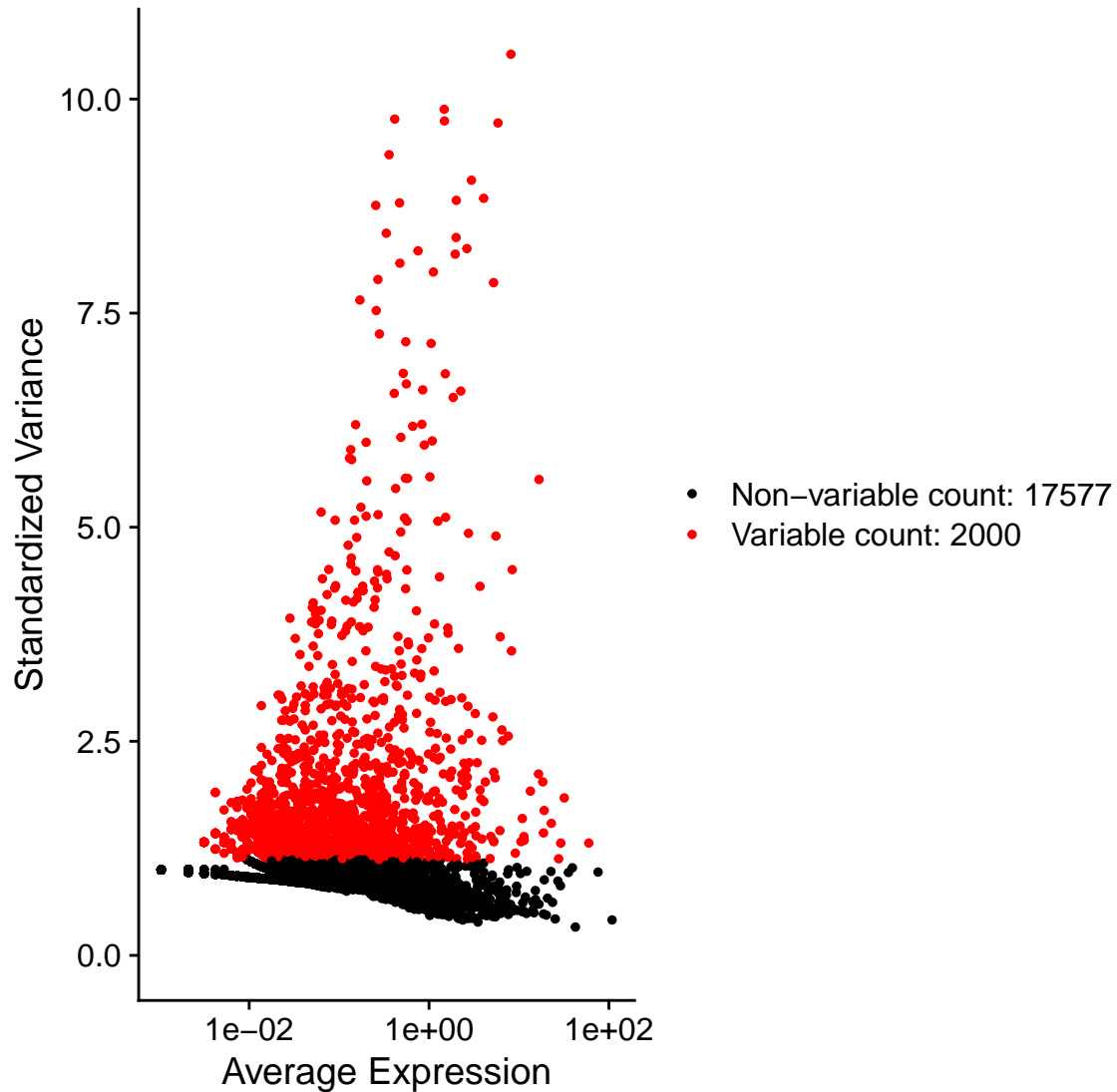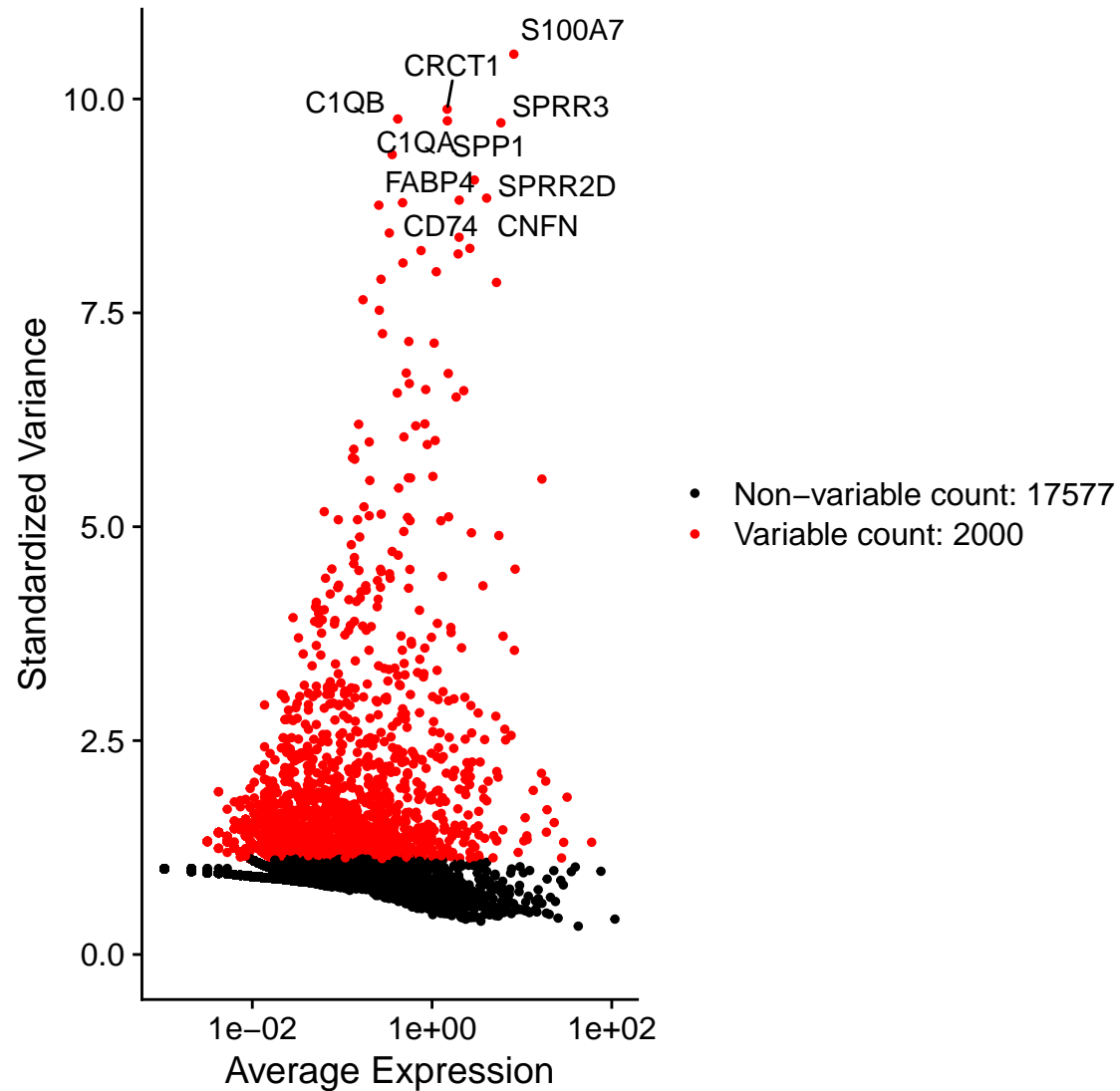

Supplement: Supplementary file 1 [file DataSheet_1.zip › raw data for editor checking/Fig2/3_Variable_Features.pdf]

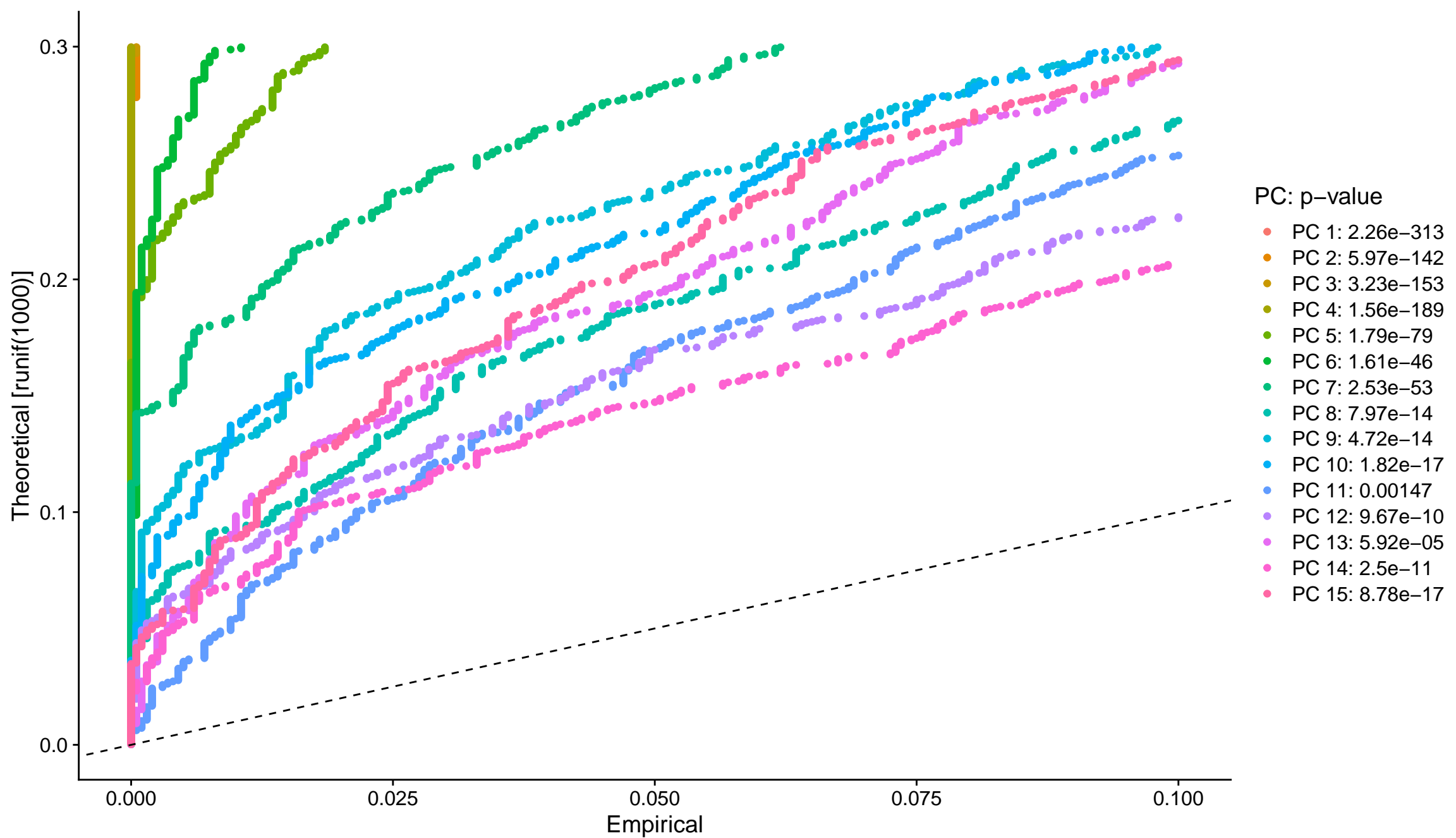

Supplement: Supplementary file 1 [file DataSheet_1.zip › raw data for editor checking/Fig2/5_jackstrawplot.pdf]

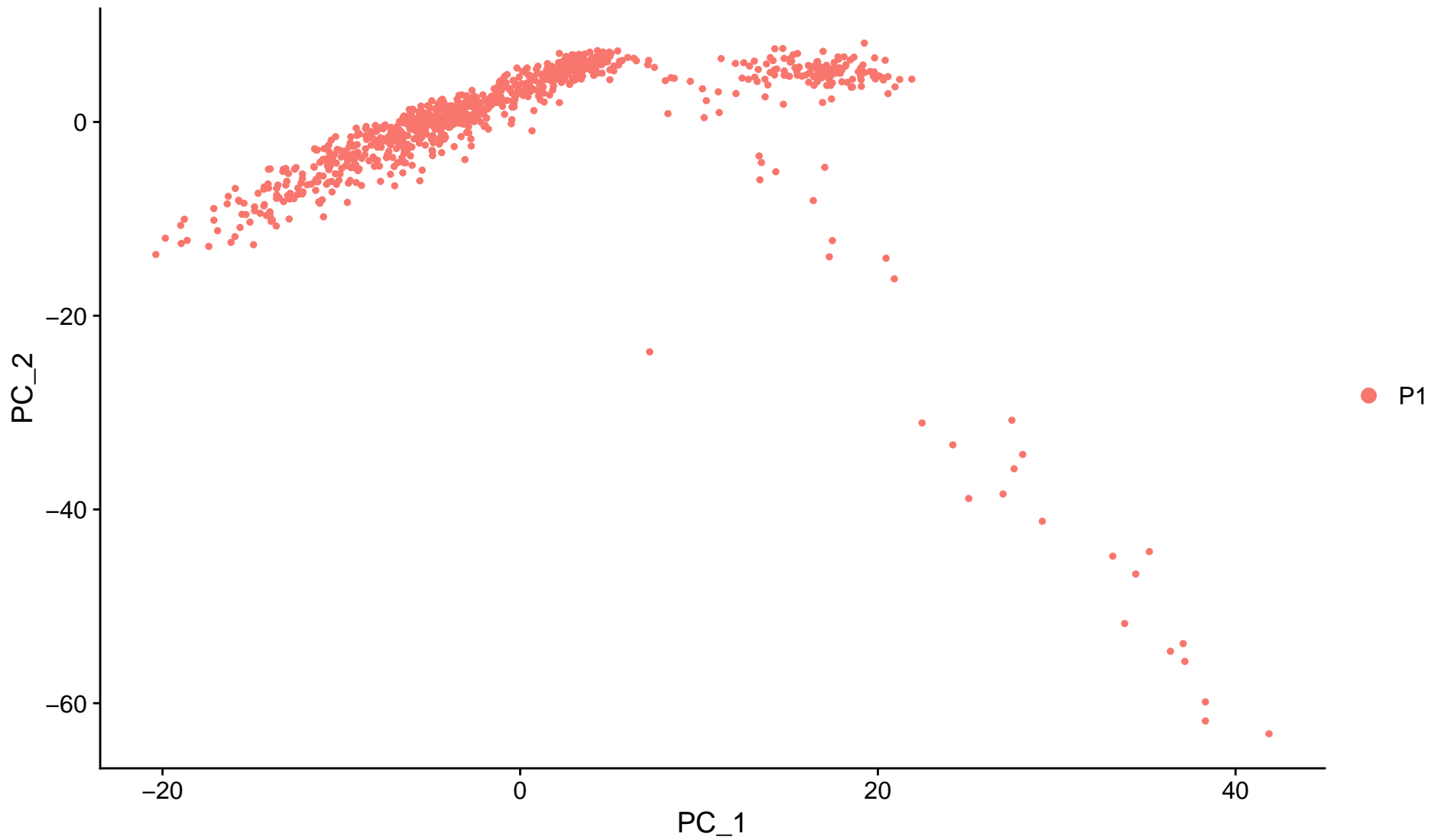

Supplement: Supplementary file 1 [file DataSheet_1.zip › raw data for editor checking/Fig2/5_PCA.pdf]

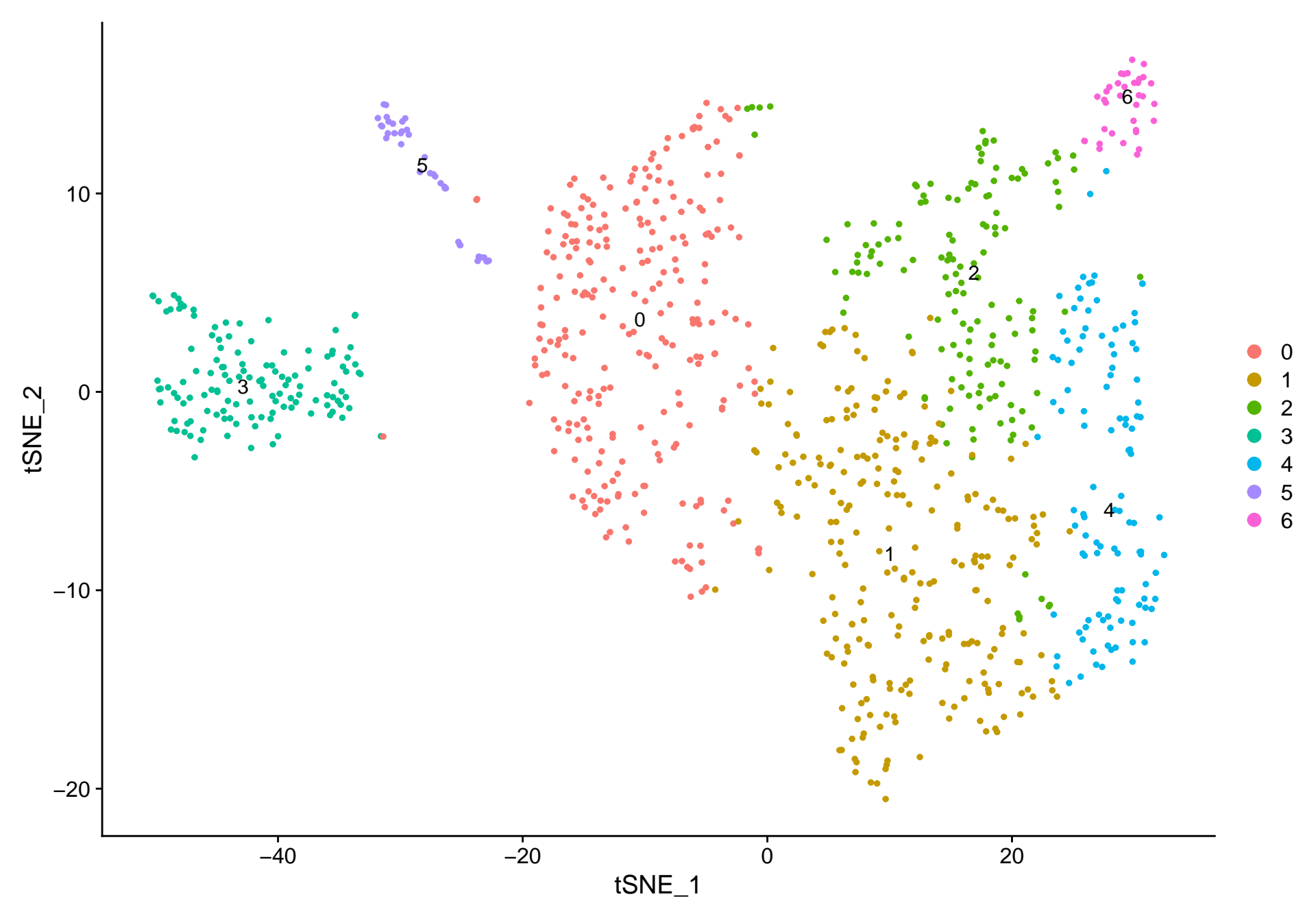

Supplement: Supplementary file 1 [file DataSheet_1.zip › raw data for editor checking/Fig2/6_tSNE.pdf]

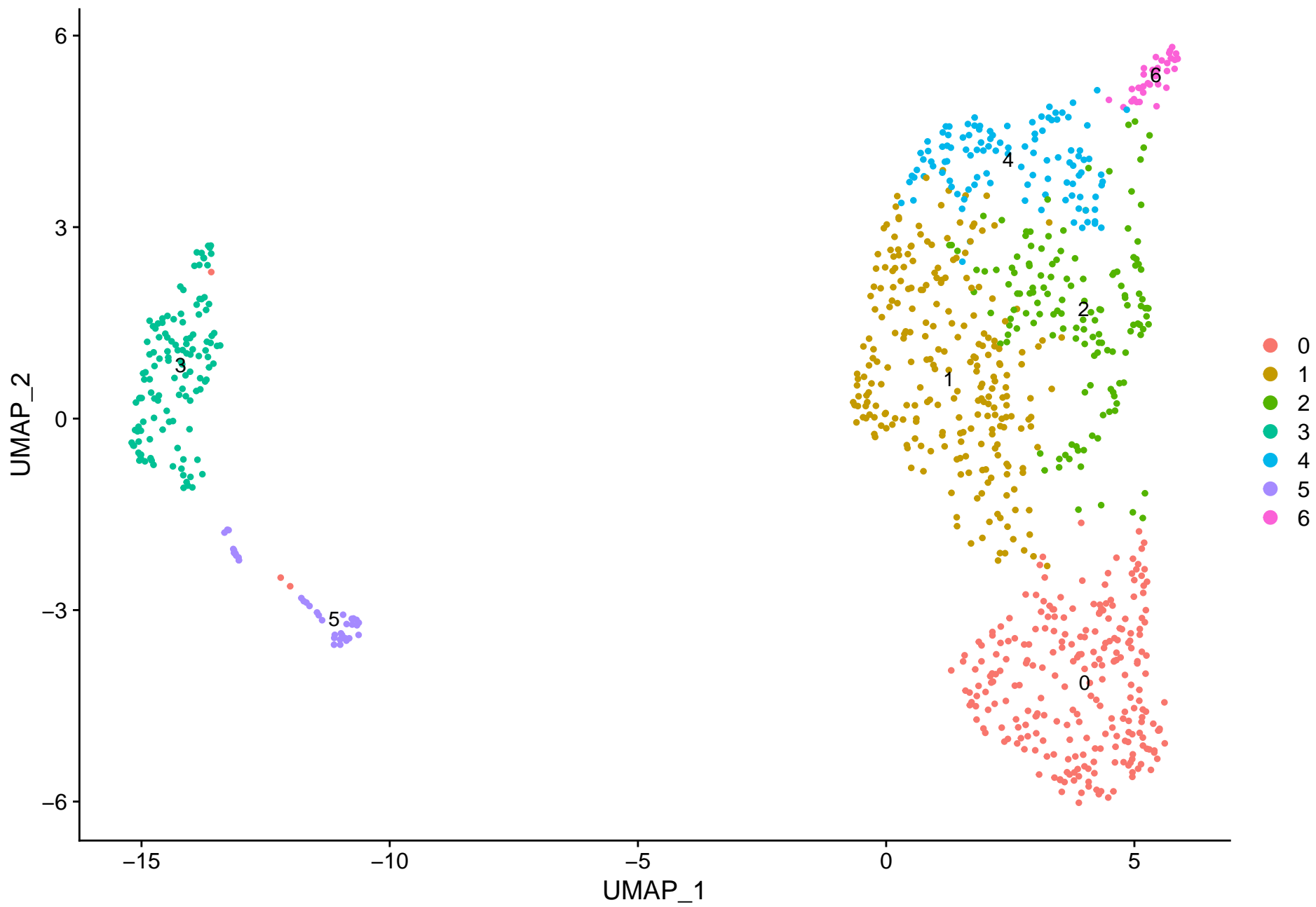

Supplement: Supplementary file 1 [file DataSheet_1.zip › raw data for editor checking/Fig2/6_UMAP.pdf]

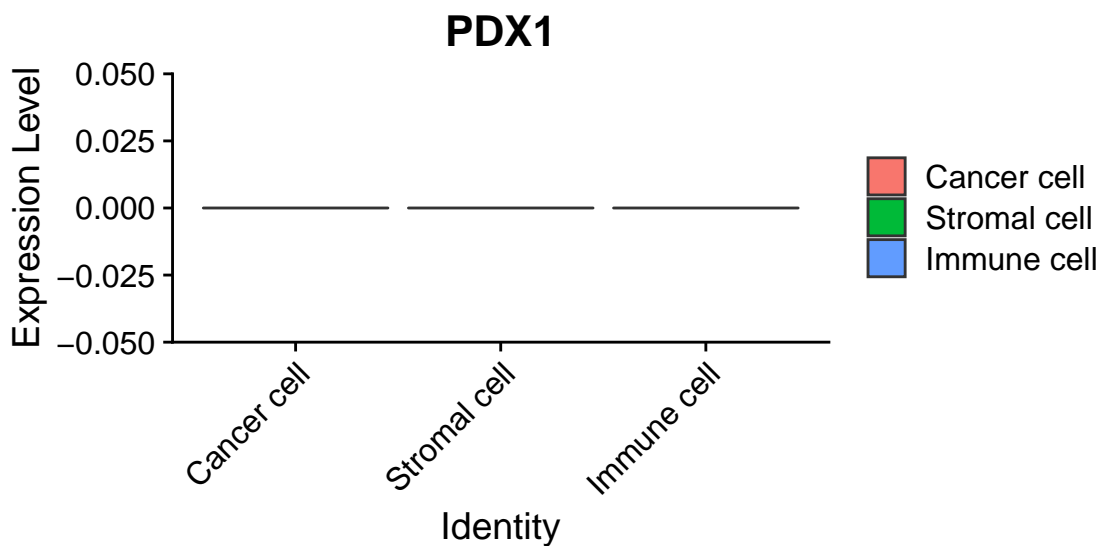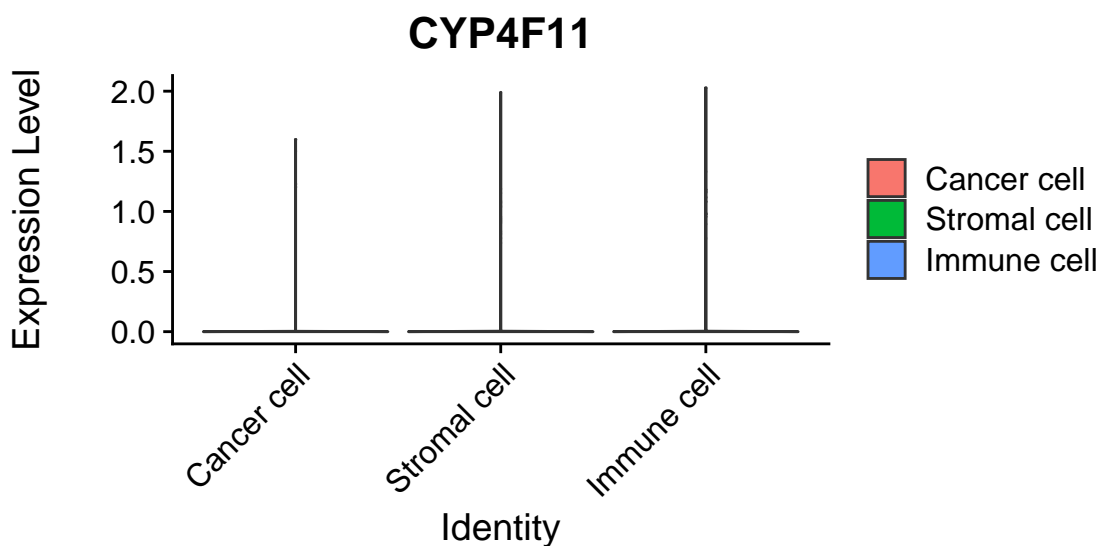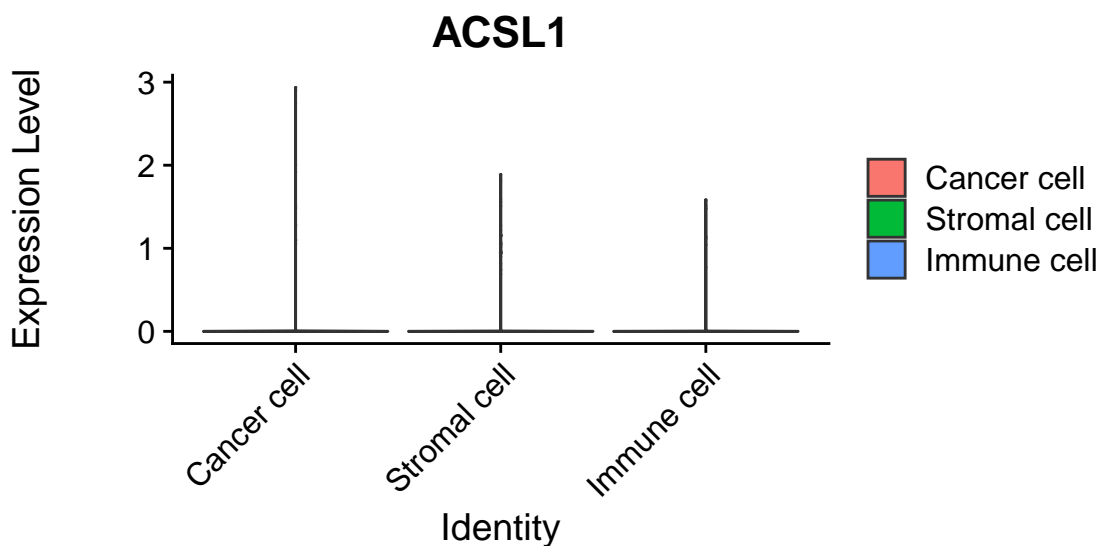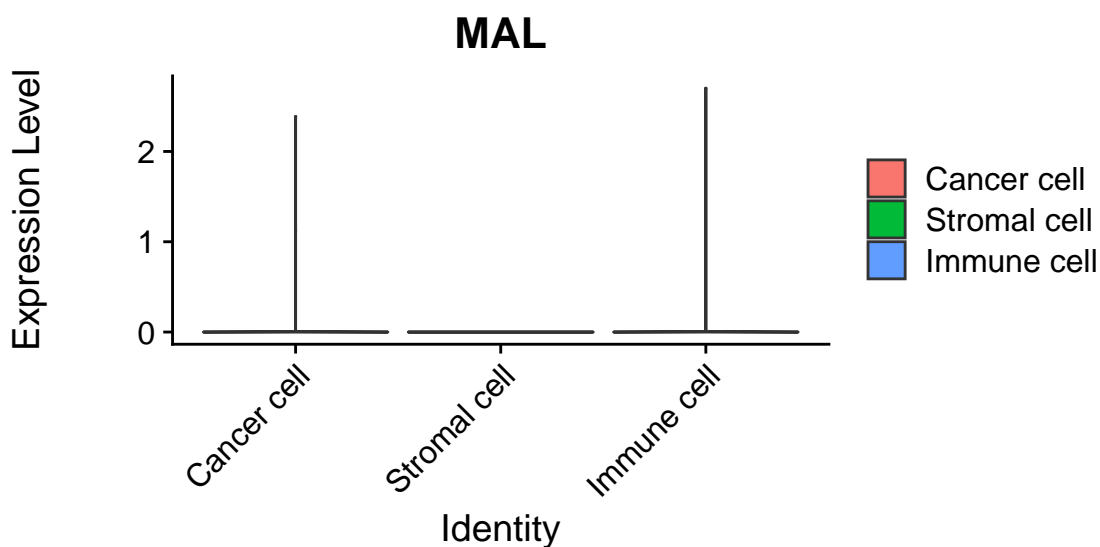

Supplement: Supplementary file 1 [file DataSheet_1.zip › raw data for editor checking/Fig2/6_violin_plot_one_1-4.pdf]

## ZNF502

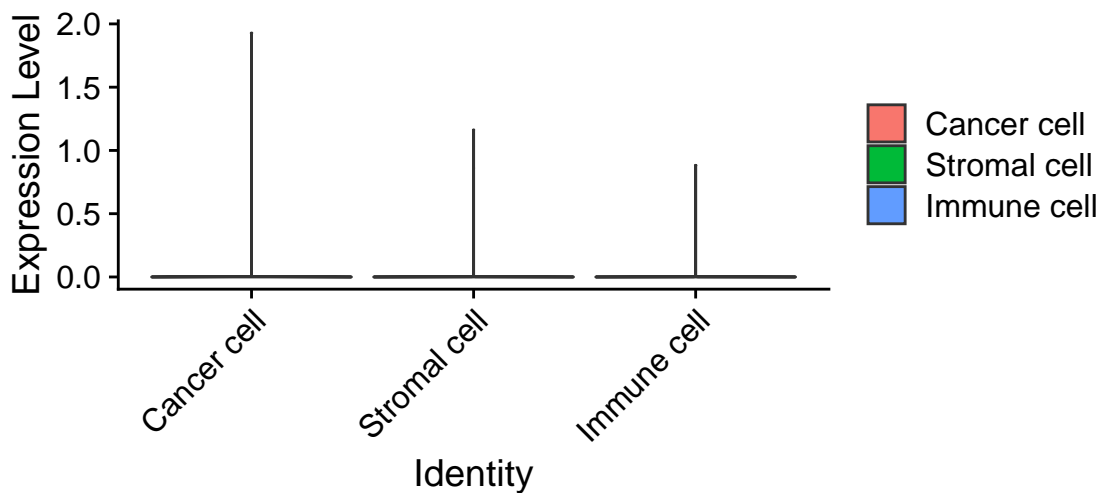

## KCNN4

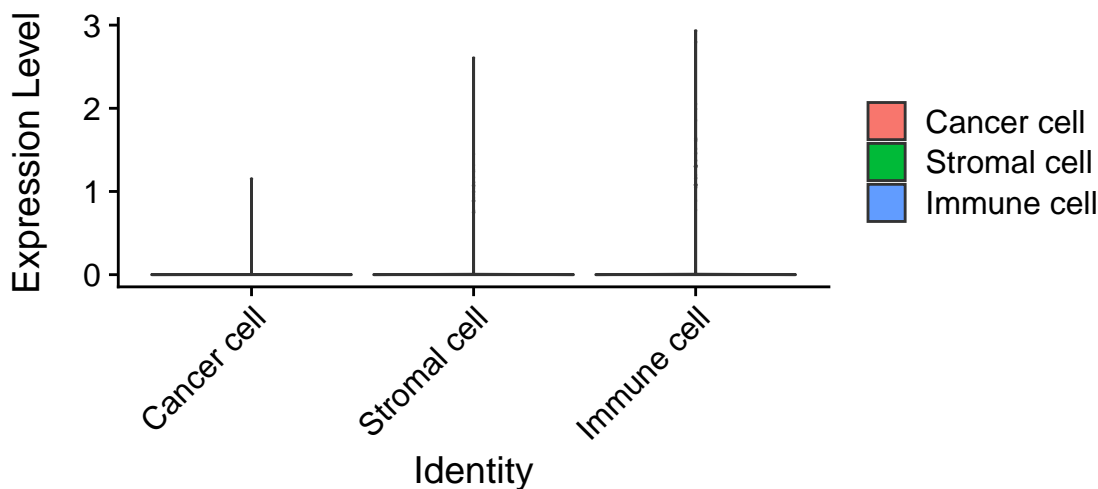

## FAM117A

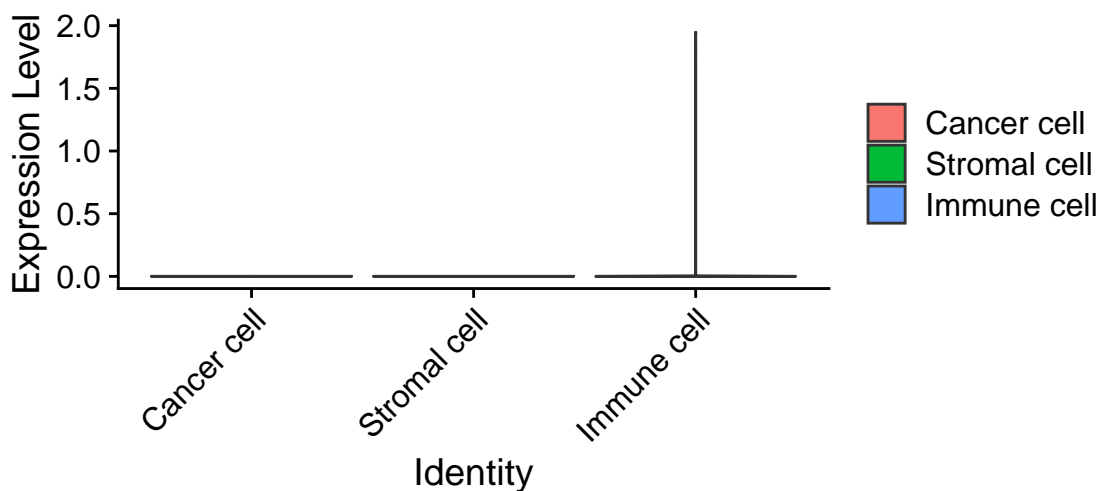

## MYEF2

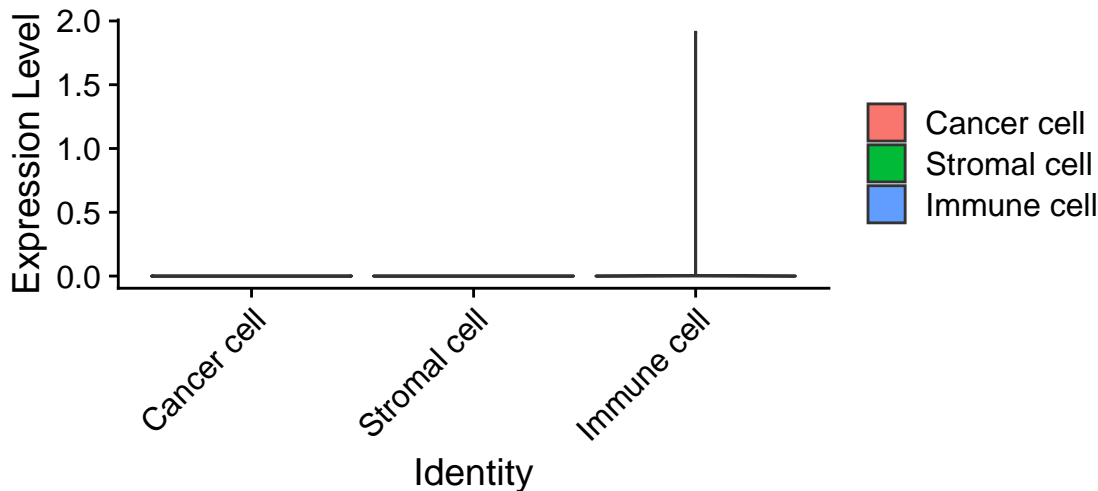

Supplement: Supplementary file 1 [file DataSheet_1.zip › raw data for editor checking/Fig2/6_violin_plot_one_5-8.pdf]

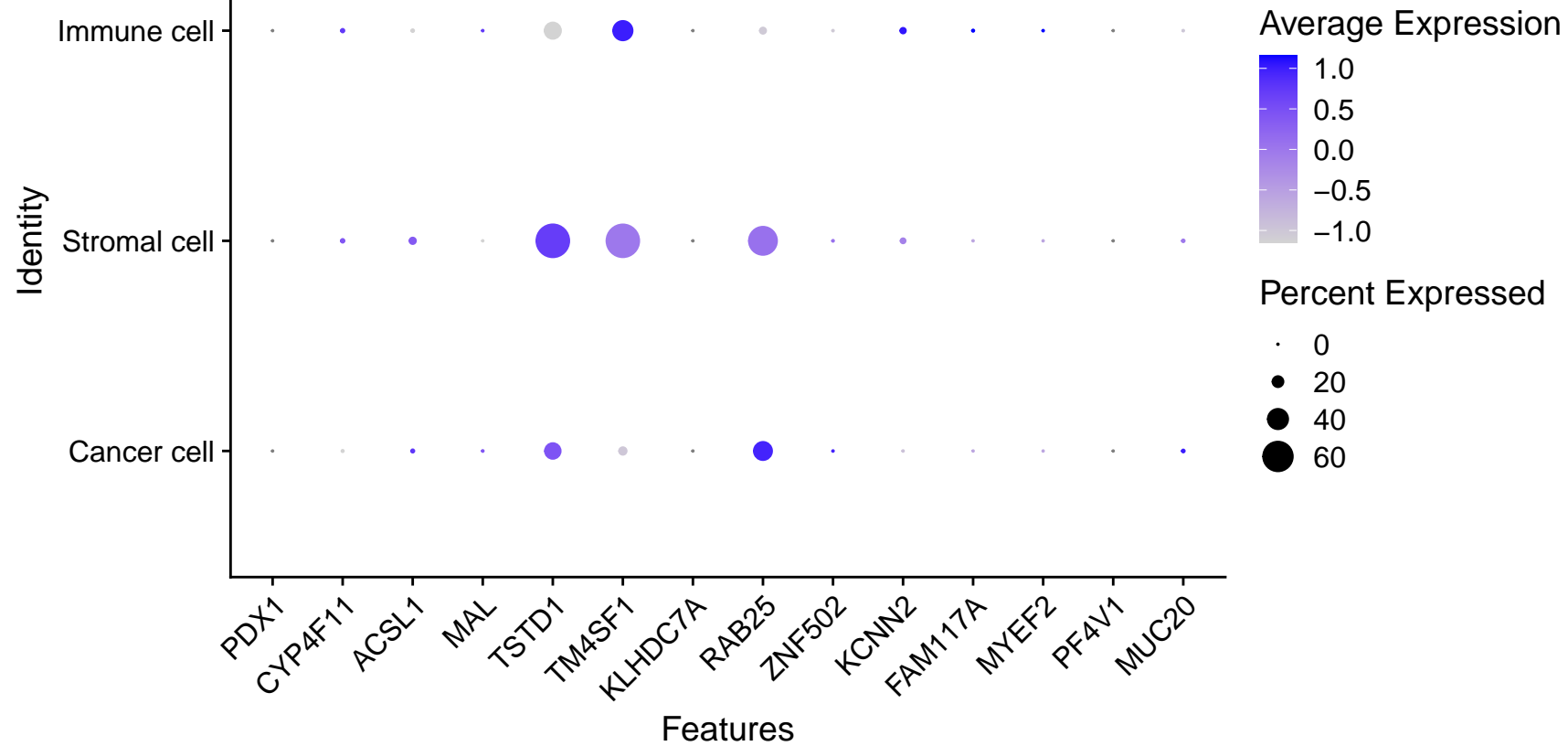

Supplement: Supplementary file 1 [file DataSheet_1.zip › raw data for editor checking/Fig2/8_markers_dotplot_celltype.pdf]

Cancer cell

Stromal cell

Immune cell

Epithelial cell

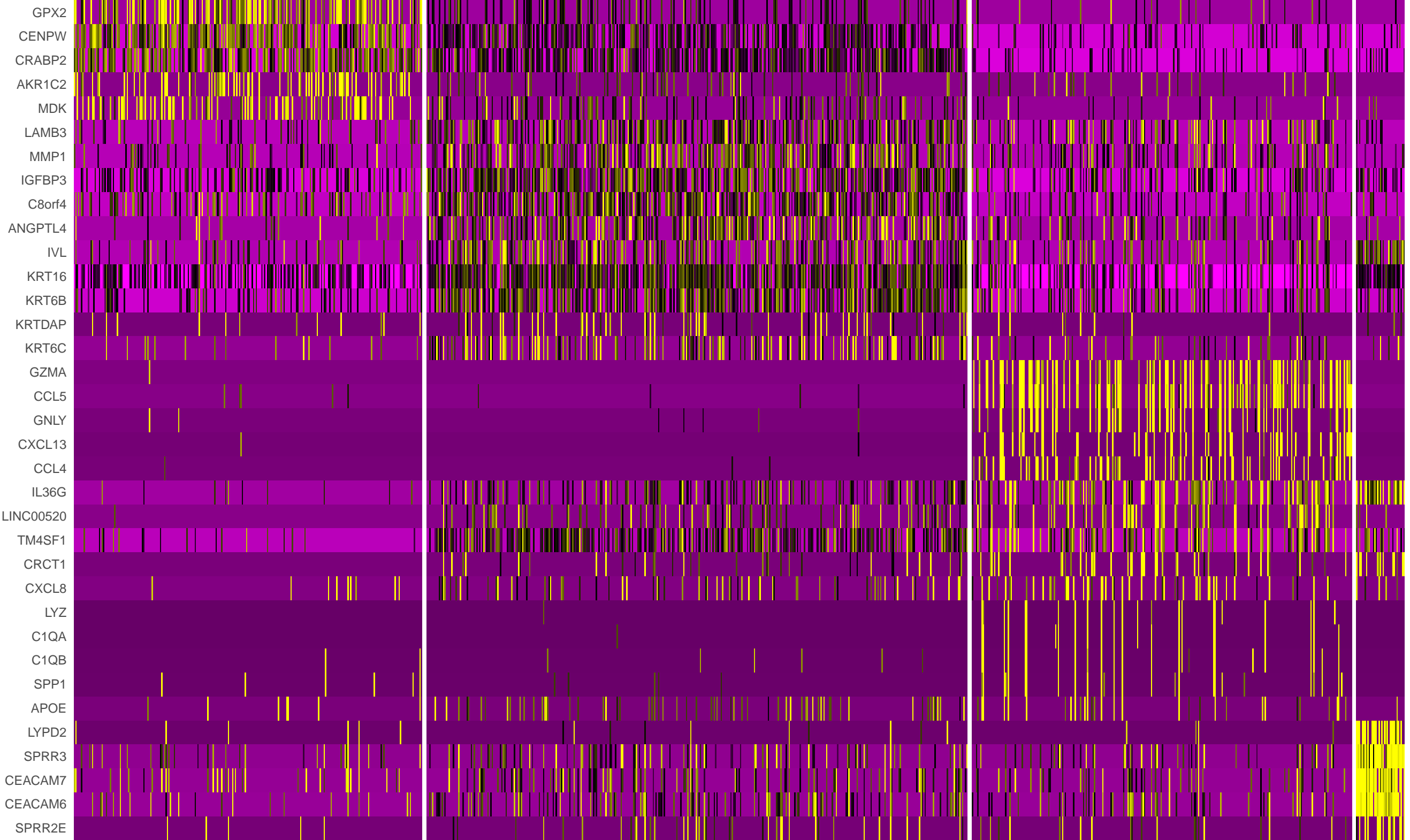

Supplement: Supplementary file 1 [file DataSheet_1.zip › raw data for editor checking/Fig2/8_markers_heatmap_celltype.pdf]

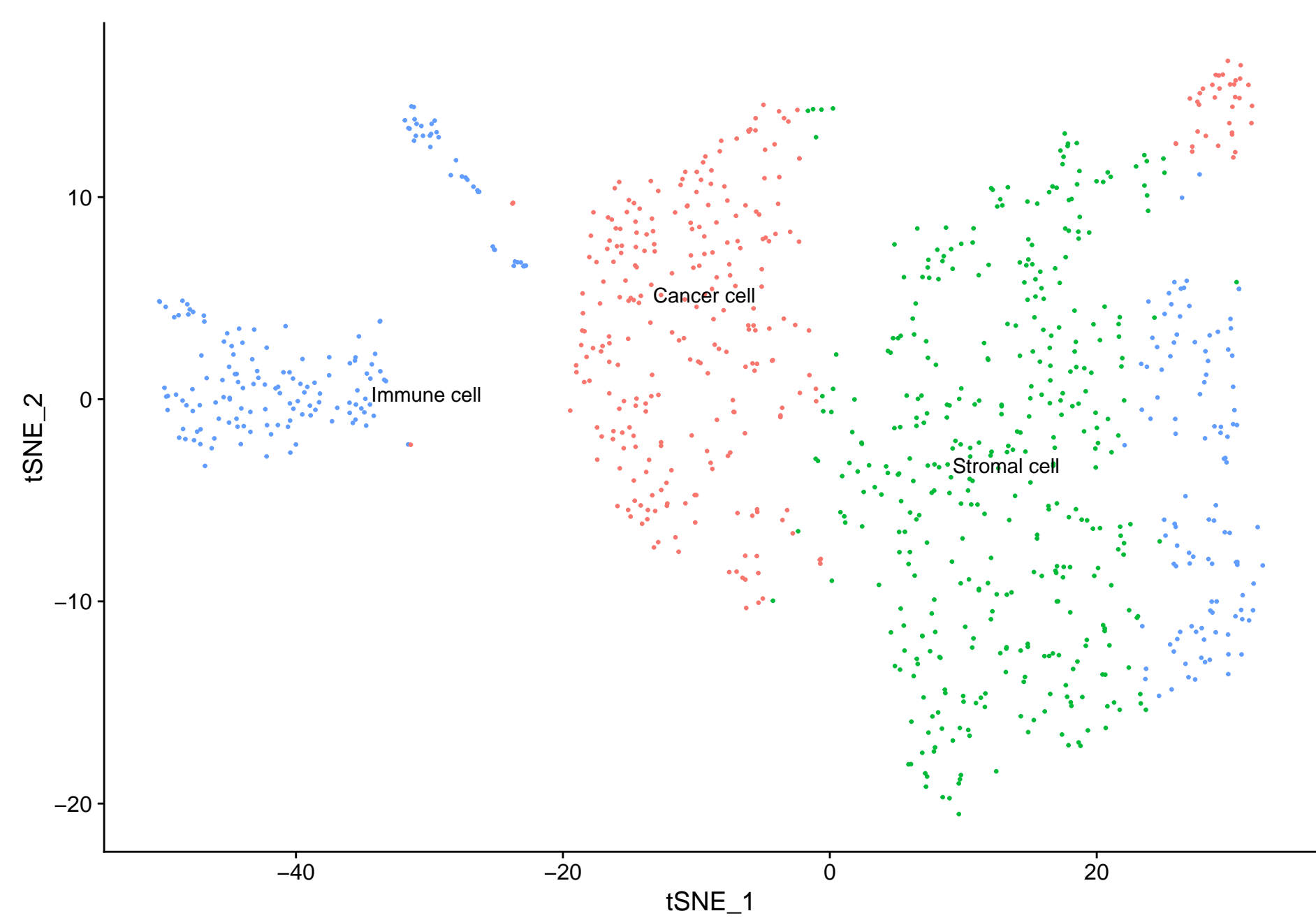

Supplement: Supplementary file 1 [file DataSheet_1.zip › raw data for editor checking/Fig2/8_umap_cell_type.pdf]

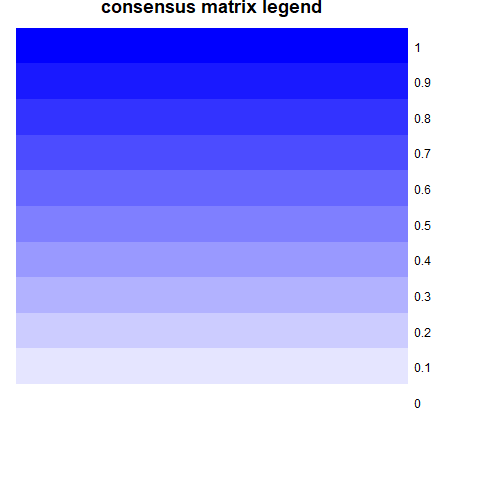

Supplement: Supplementary file 1 [file DataSheet_1.zip › raw data for editor checking/Fig3/consensus001.png]

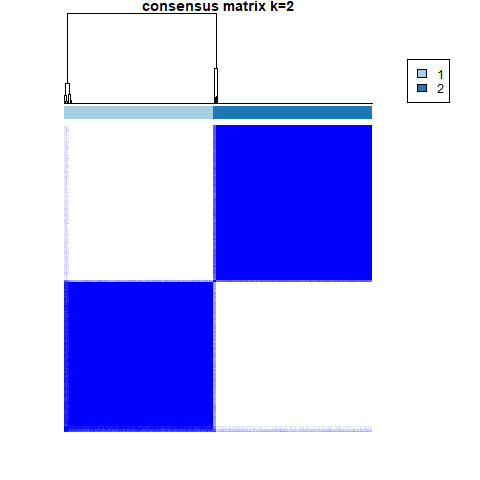

Supplement: Supplementary file 1 [file DataSheet_1.zip › raw data for editor checking/Fig3/consensus002.png]

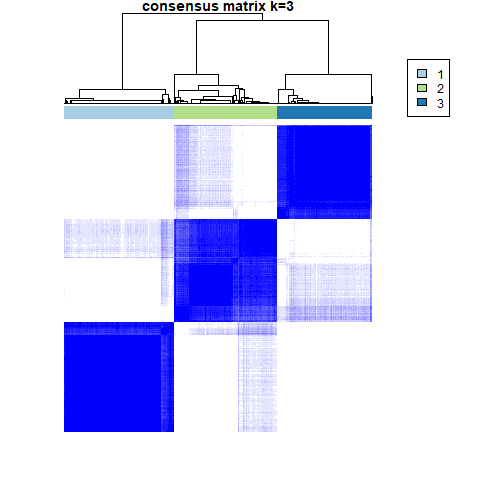

Supplement: Supplementary file 1 [file DataSheet_1.zip › raw data for editor checking/Fig3/consensus003.png]

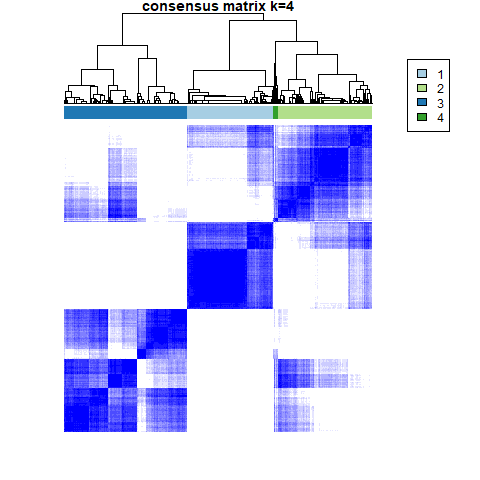

Supplement: Supplementary file 1 [file DataSheet_1.zip › raw data for editor checking/Fig3/consensus004.png]

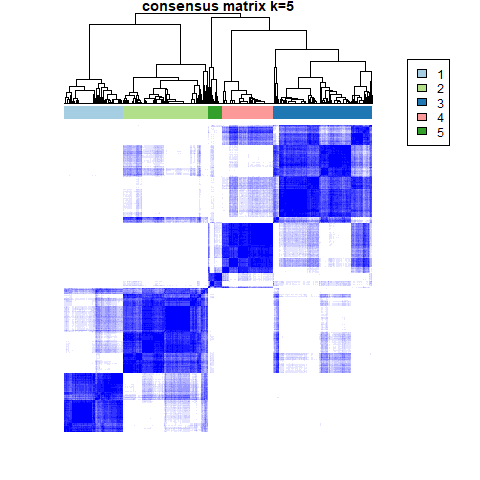

Supplement: Supplementary file 1 [file DataSheet_1.zip › raw data for editor checking/Fig3/consensus005.png]

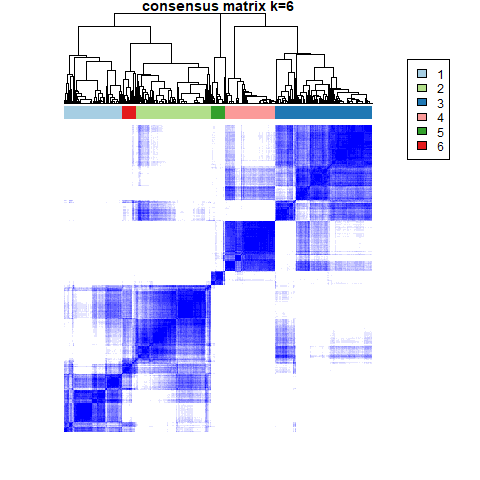

Supplement: Supplementary file 1 [file DataSheet_1.zip › raw data for editor checking/Fig3/consensus006.png]

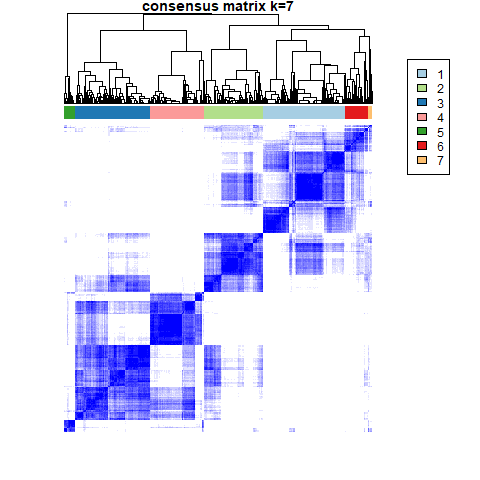

Supplement: Supplementary file 1 [file DataSheet_1.zip › raw data for editor checking/Fig3/consensus007.png]

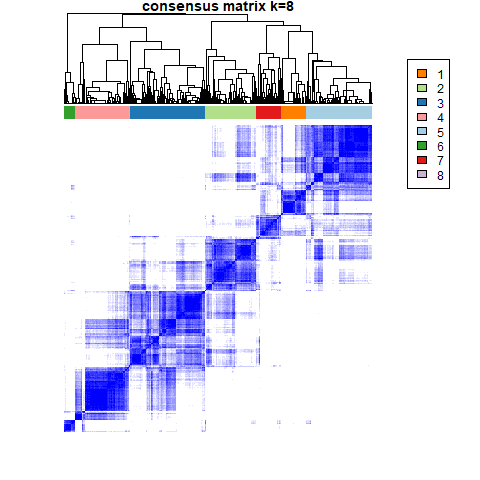

Supplement: Supplementary file 1 [file DataSheet_1.zip › raw data for editor checking/Fig3/consensus008.png]

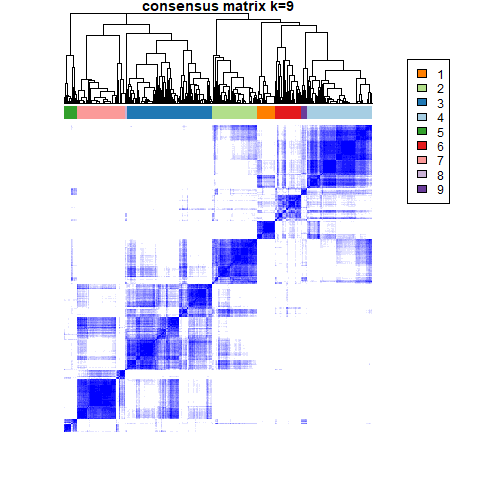

Supplement: Supplementary file 1 [file DataSheet_1.zip › raw data for editor checking/Fig3/consensus009.png]

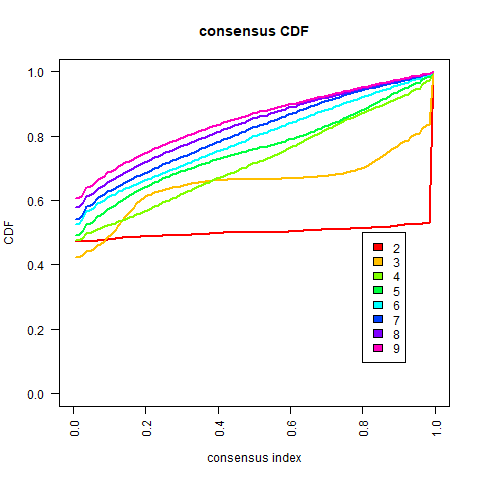

Supplement: Supplementary file 1 [file DataSheet_1.zip › raw data for editor checking/Fig3/consensus010.png]

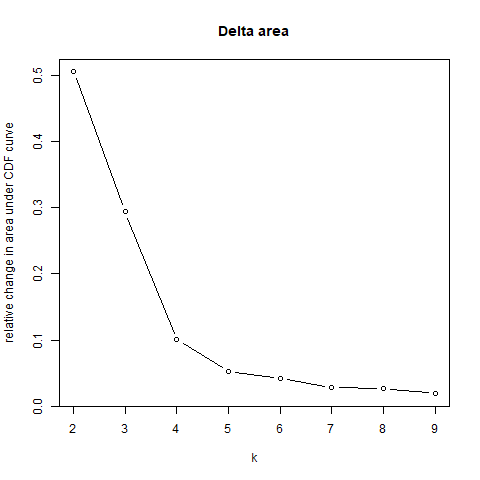

Supplement: Supplementary file 1 [file DataSheet_1.zip › raw data for editor checking/Fig3/consensus011.png]

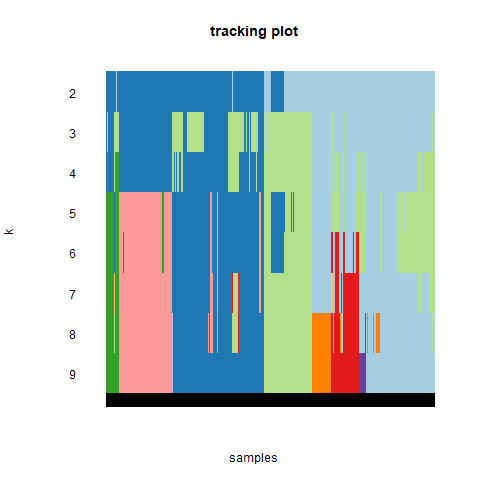

Supplement: Supplementary file 1 [file DataSheet_1.zip › raw data for editor checking/Fig3/consensus012.png]

Response 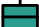 CR/PR 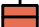 SD/PD

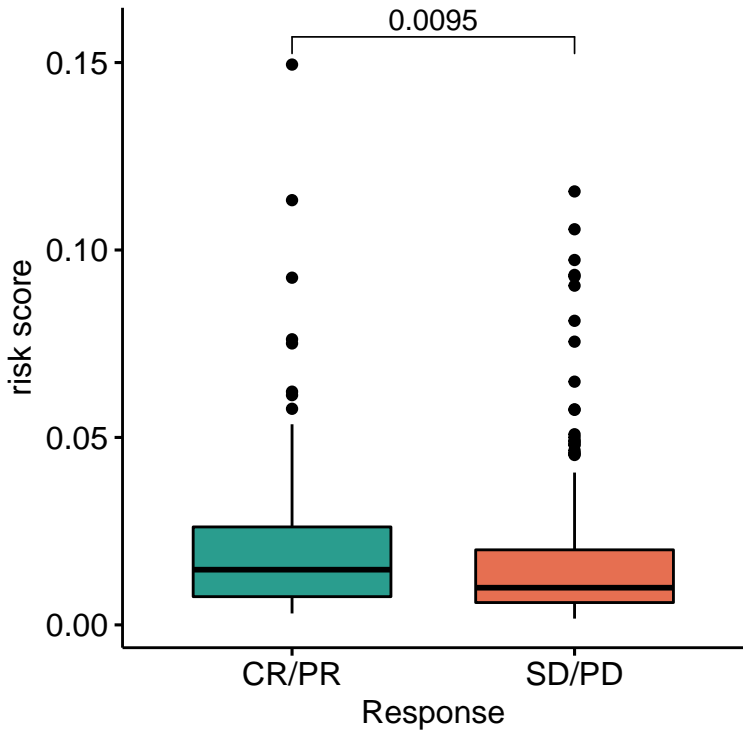

Supplement: Supplementary file 1 [file DataSheet_1.zip › raw data for editor checking/Fig7-Fig8/Response.pdf]

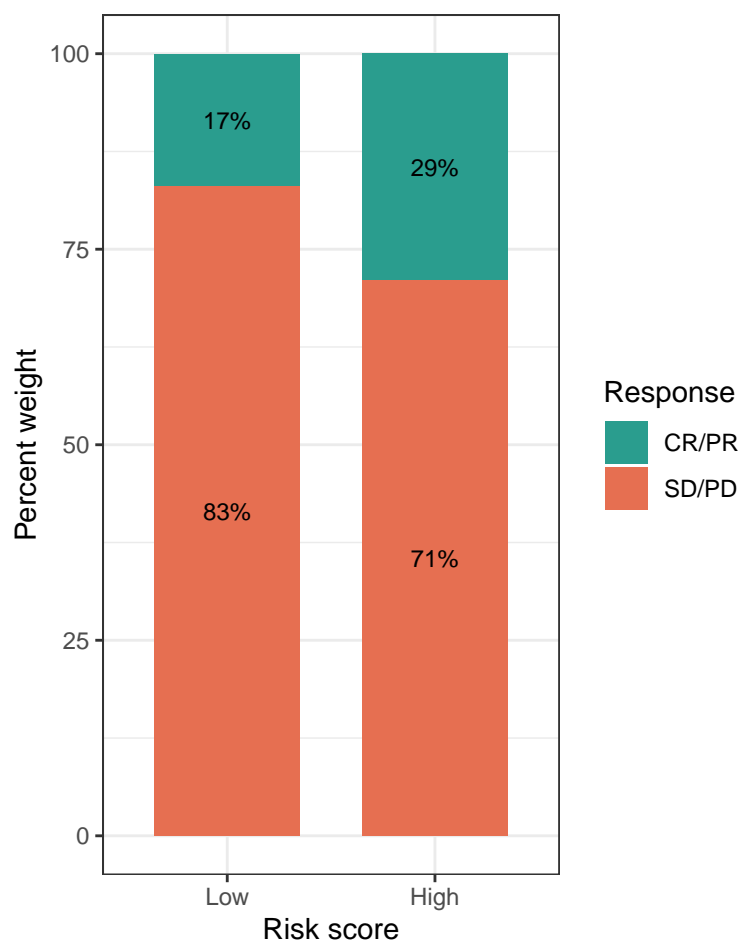

Supplement: Supplementary file 1 [file DataSheet_1.zip › raw data for editor checking/Fig7-Fig8/Responsebarplot.pdf]

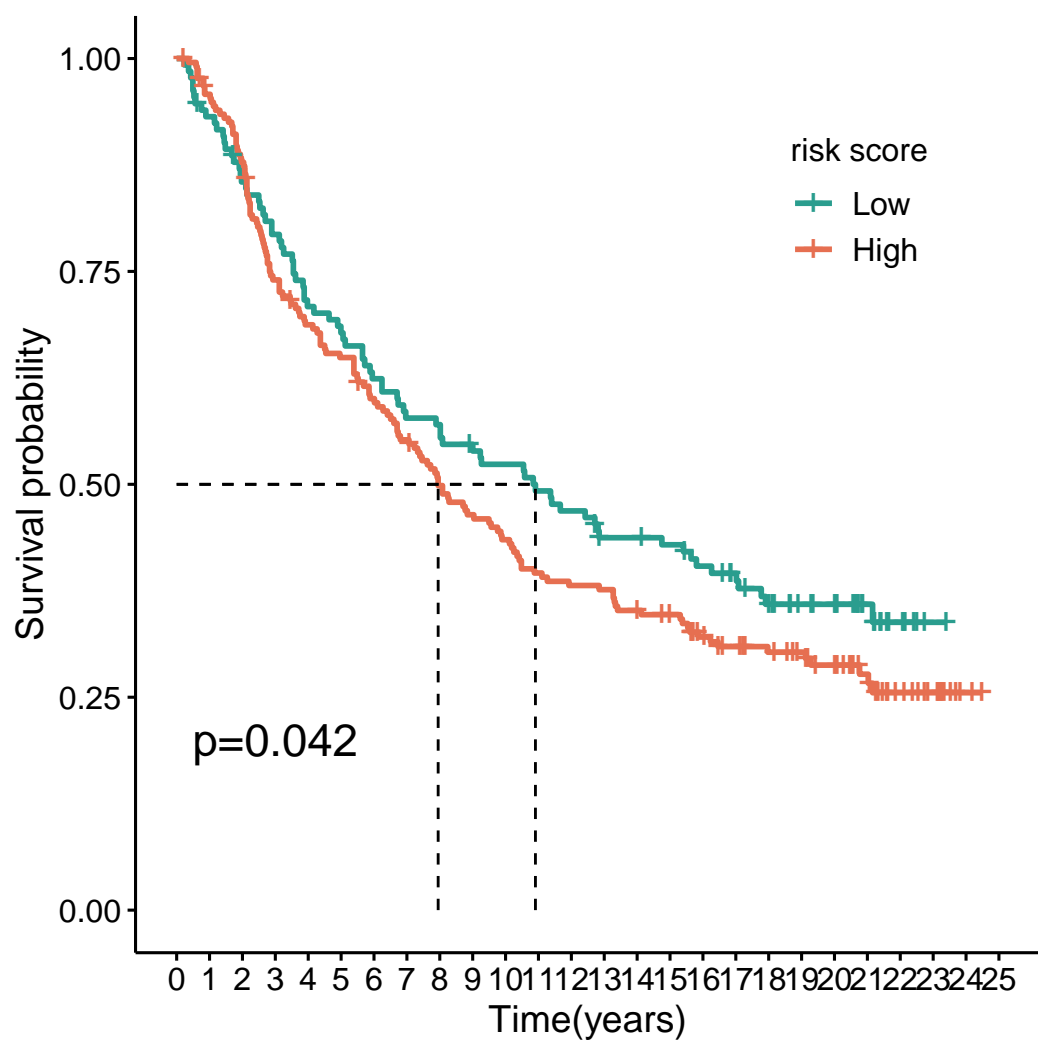

Supplement: Supplementary file 1 [file DataSheet_1.zip › raw data for editor checking/Fig7-Fig8/survival.pdf]

**A**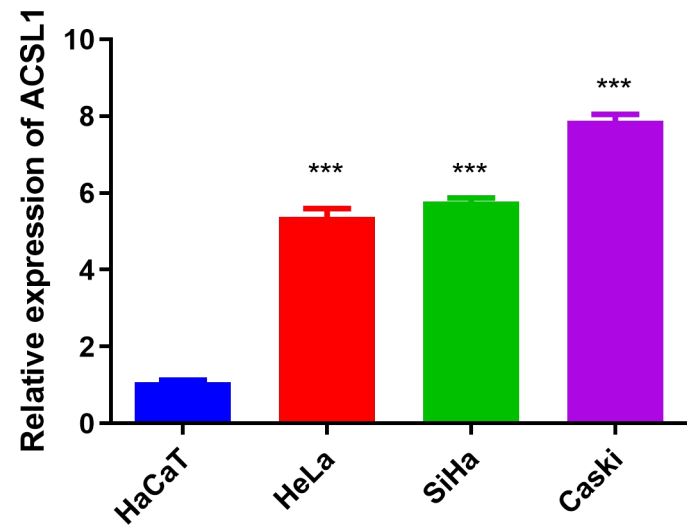**B**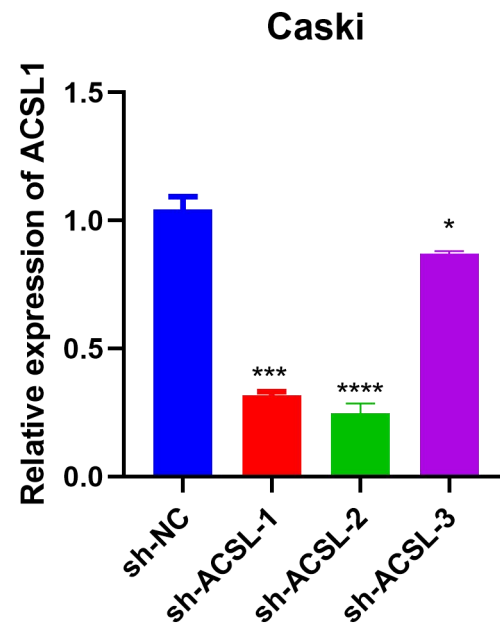**C**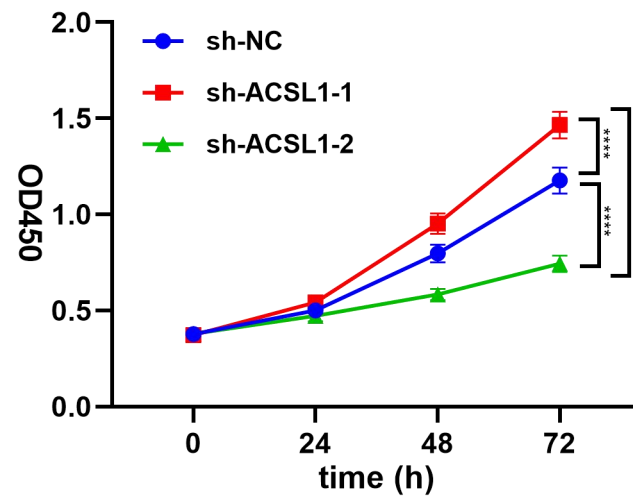

Supplement: Supplementary file 1 [file DataSheet_1.zip › raw data for editor checking/FigS1/FigureS1.pdf]

## Slide 1
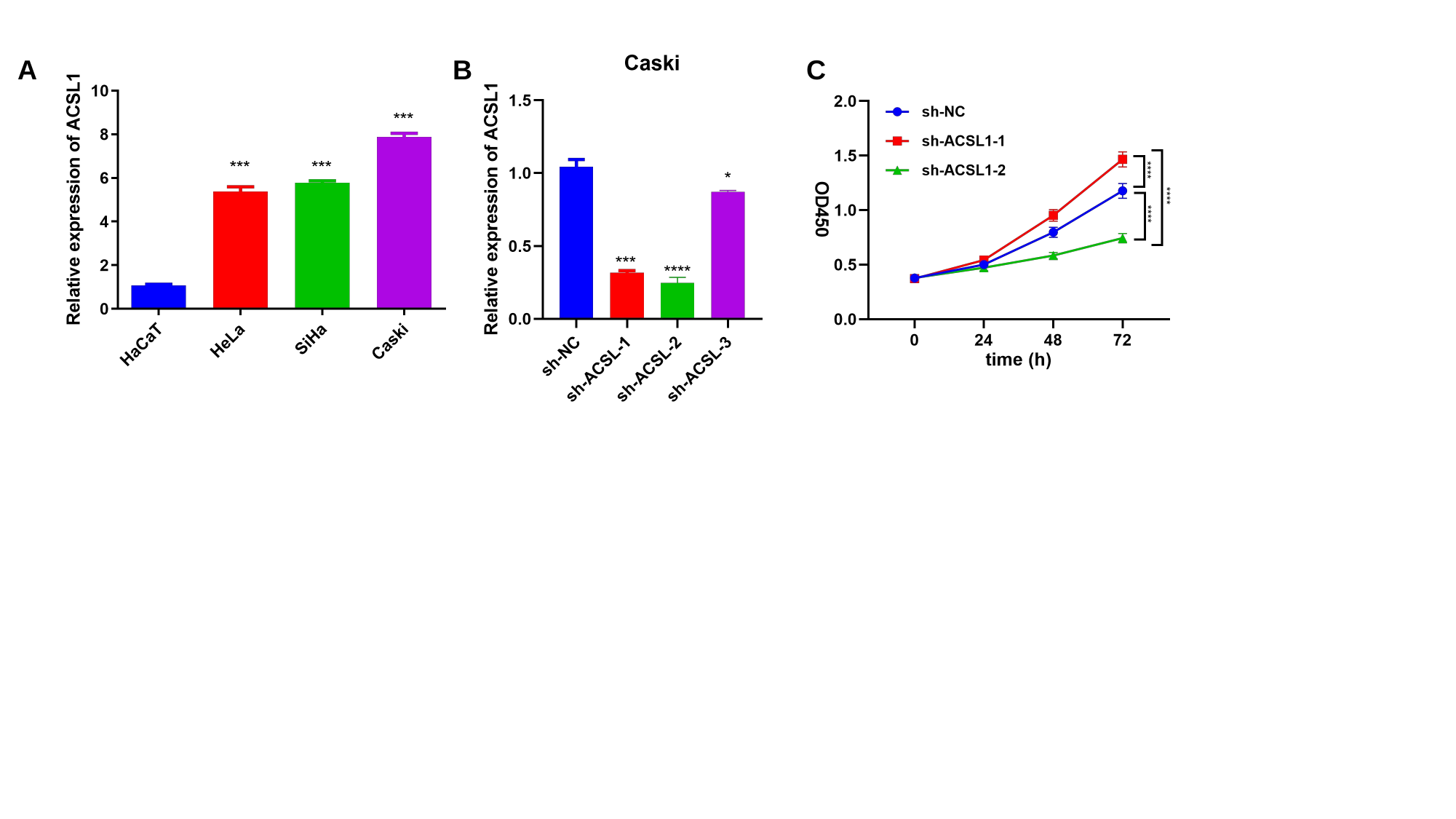

A
B
C

Supplement: Supplementary file 1 [file DataSheet_1.zip › raw data for editor checking/FigS1/FigureS1.pptx]
