# Supplementary material for: A novel cervix carcinoma biomarker: Pathological-epigenomics, integrated analysis of MethylMix algorithm and pathology for predicting response to cancer immunotherapy
Source: Front Oncol. 2022 Nov 2;12:1053800. doi: 10.3389/fonc.2022.1053800 (PMC9667097; doi:10.3389/fonc.2022.1053800)
Supplement: Supplementary file 2 [file DataSheet_2.docx]

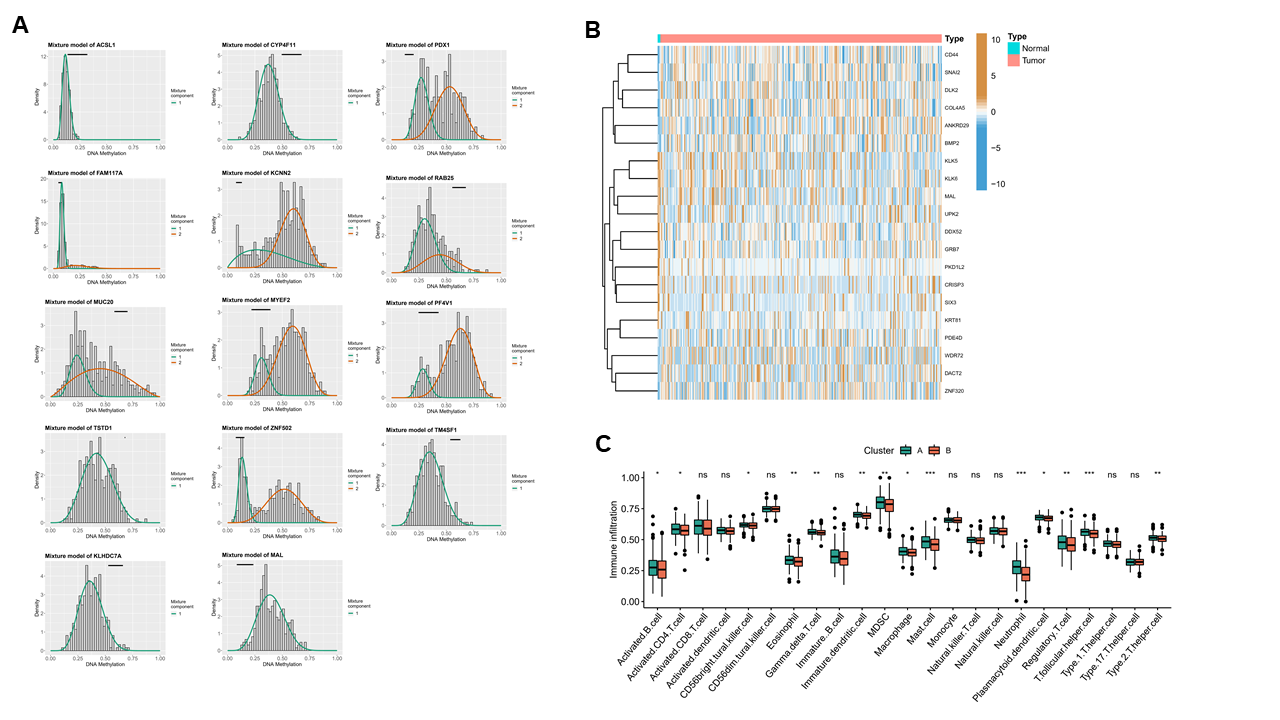


**Supplementary Figure 1. Overview of methylation driven genes in cervical cancer.**

(A) Representative distribution map of MDGs, reflecting the distribution of methylation values. (B) Heat map of 20 methylation driven genes in cervical cancer. (C) Infiltration levels of 22 cell subsets among different methylation-driven subtypes.


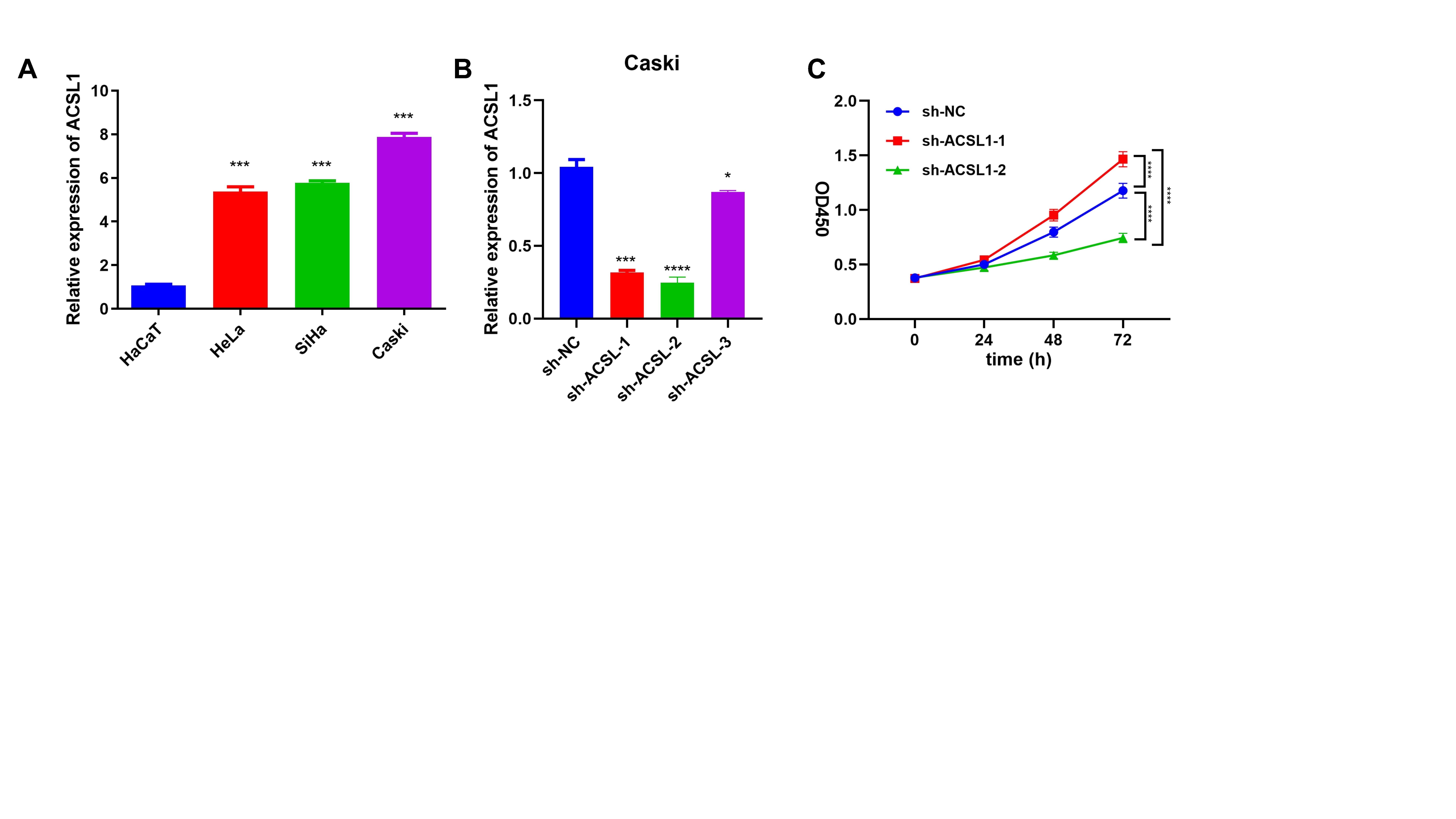
**Supplementary Figure 2.** **In vitro assays.**

(A) The expression of ACSL1 of cell line (HaCaT，HeLa，SiHa，Caski) were detected by qRT-PCR. (B) Measurement of transfection efficiency. (C) The proliferation of Caski cells was examined by CCK-8 assay.


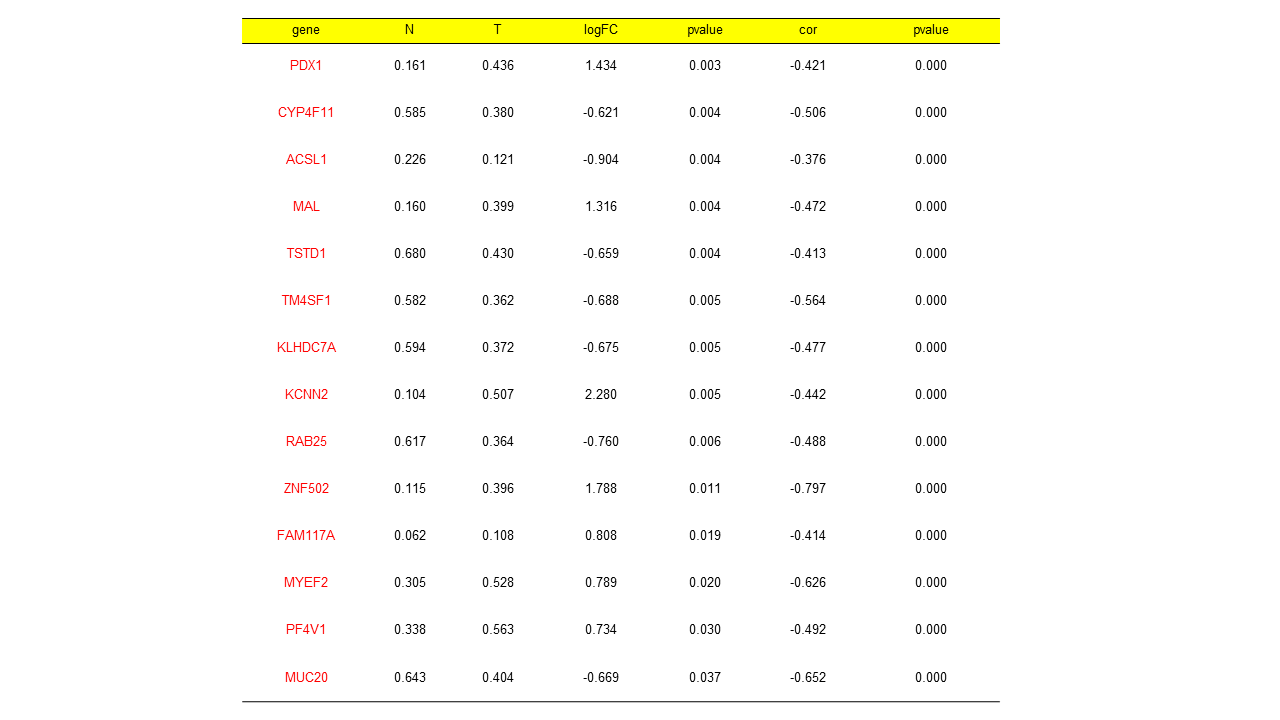
**Table S1.** Methylation driven genes in cervical cancer.
